# *VRN1* genes variability in tetraploid wheat species with a spring growth habit

**DOI:** 10.1186/s12870-016-0924-z

**Published:** 2016-11-16

**Authors:** Irina Konopatskaia, Valeriya Vavilova, Elena Ya. Kondratenko, Alexandr Blinov, Nikolay P. Goncharov

**Affiliations:** 1 The Federal Research Center Institute of Cytology and Genetics SB RAS, Prospekt Lavrentyeva, 10, Novosibirsk, 630090 Russian Federation; 2 Novosibirsk State University, Pirogova 2, 630090 Novosibirsk, Russian Federation; 3 Novosibirsk State Agrarian University, Dobrolubov Str., 160, Novosibirsk, 630039 Russian Federation

**Keywords:** Evolution, Growth habit, *Triticum*, vernalization, *VRN1* gene, Wheat

## Abstract

**Background:**

Vernalization genes *VRN1* play a major role in the transition from vegetative to reproductive growth in wheat. In di-, tetra- and hexaploid wheats the presence of a dominant allele of at least one *VRN1* gene homologue (*Vrn-A1*, *Vrn-B1*, *Vrn-G1* or *Vrn-D1*) determines the spring growth habit. Allelic variation between the *Vrn-1* and *vrn-1* alleles relies on mutations in the promoter region or the first intron. The origin and variability of the dominant *VRN1* alleles, determining the spring growth habit in tetraploid wheat species have been poorly studied.

**Results:**

Here we analyzed the growth habit of 228 tetraploid wheat species accessions and 25 % of them were spring type. We analyzed the promoter and first intron regions of *VRN1* genes in 57 spring accessions of tetraploid wheats. The spring growth habit of most studied spring accessions was determined by previously identified dominant alleles of *VRN1* genes. Genetic experiments proof the dominant inheritance of *Vrn-A1d* allele which was widely distributed across the accessions of *Triticum dicoccoides*. Two novel alleles were discovered and designated as *Vrn-A1b.7* and *Vrn-B1dic. Vrn-A1b.7* had deletions of 20 bp located 137 bp upstream of the start codon and mutations within the VRN-box when compared to the recessive allele of *vrn-A1*. So far the *Vrn-A1d* allele was identified only in spring accessions of the *T. dicoccoides* and *T. turgidum* species. *Vrn-B1dic* was identified in *T. dicoccoides* IG46225 and had 11 % sequence dissimilarity in comparison to the promoter of *vrn-B1*. The presence of *Vrn-A1b.7* and *Vrn-B1dic* alleles is a predicted cause of the spring growth habit of studied accessions of tetraploid species. Three spring accessions *T. aethiopicum* K-19059, *T. turanicum* K-31693 and *T. turgidum* cv. Blancal possess recessive alleles of both *VRN-A1* and *VRN-B1* genes*.* Further investigations are required to determine the source of spring growth habit of these accessions.

**Conclusions:**

New allelic variants of the *VRN-A1* and *VRN-B1* genes were identified in spring accessions of tetraploid wheats. The origin and evolution of *VRN-A1* alleles in di- and tetraploid wheat species was discussed.

**Electronic supplementary material:**

The online version of this article (doi:10.1186/s12870-016-0924-z) contains supplementary material, which is available to authorized users.

## Background

Flowering time is a critical agronomical trait that has a major impact on the adaptation to local climate and environmental conditions and grain yield in wheat species. Wheat cultivars differ in their requirements for extended exposure to low-temperature (vernalization) to initiate the transition from vegetative growth to flowering [1]. Ancestors of wheat, as well as modern wheat species with a winter growth habit, are planted in autumn and flower during the subsequent spring. These species require vernalization for transition from vegetative to reproductive growth. The vernalization requirement prevents the fragile flower meristems from being damaged by low temperatures and ensures that flowering occurs under optimal conditions in spring. Modern wheat cultivars with spring growth habit lack this vernalization requirement and can be planted in spring [2].

Vernalization-induced flowering in wheat is mainly controlled by the vernalization genes *VRN1*, *VRN2*, *VRN3* and *VRN4* which interact with each other as well as other flowering control pathways [1–8]. *VRN1* genes mapped to the long arms of the 5 homoeological group chromosomes play a central role in complex vernalization pathways [9–12]. The floral activator *VRN1* encodes a MADS-box transcription factor that is required for the initiation of reproductive development at the shoot apical meristem [3, 13, 14]. The expression of *VRN1* occurs at a low basal level but a measurable increase is seen during prolonged treatment with low temperatures [3, 13, 14].

In di-, tetra- and hexaploid wheats the presence of a dominant allele of at least one *VRN1* gene homologue (*Vrn-A1*, *Vrn-B1*, *Vrn-G1* or *Vrn-D1*) determines the spring growth habit. Allelic variation between the *Vrn-1* and *vrn-1* alleles relies on mutations in the promoter region or the first intron.

Analysis of diploid wheat species revealed three recessive alleles *vrn-A* ^*m*^ *1*, *vrn-A1u* and *vrn-A* ^*m*^ *1b* which determine the winter growth habit [3, 15–18]. The *vrn-A* ^*m*^ *1* allele is distributed in all diploid wheat species and represents the only allelic variant so far identified in *Triticum sinskajae* A. Filat. et Kurk. [3, 15–18]. The *vrn-A1u* of *T. urartu* Thum. ex Gandil. is identical to *vrn-A1* of winter accessions of polyploid wheats and differs from *vrn-A* ^*m*^ *1* by a short deletion in the promoter [16, 17]. The *vrn-A* ^*m*^ *1b* with a 48-bp deletion in the *VRN1* promoter in compare to *vrn-A* ^*m*^ *1* so far was found only in accessions of *T. monococcum* L. [3, 18]. In diploid wheat *T. monococcum* several dominant *Vrn-A1* alleles which possess variable mutations in the promoter and/or first intron region were identified [3, 15–18]. Two dominant alleles from diploid wild wheat *T. boeoticum* Boiss. *Vrn-A* ^*m*^ *1f* and *Vrn-A* ^*m*^ *1a* (*Vrn-A1h*) posses short deletions in the promoter region [16, 17]. No dominant alleles were identified in *T. urartu* [16, 17].

In tetraploid wheat species of sections *Timopheevii* A. Filat. et Dorof. and *Dicoccoides* Flaksb. five recessive alleles of *VRN1* genes (*vrn-A1*(*vrn-A1u*), *vrn-A1b.3*, *vrn-A1b.4*, *vrn-B1* and *vrn-G1*) were identified [16, 17, 19]. The sequences of *vrn-B1* and *vrn-G1* alleles were identical to each other [16]. Several dominant and recessive alleles of *VRN-A1*, *VRN-B1* and *VRN-G1* genes were identified in tetraploid wheat of two sections *Timopheevii* A. Filat. et Dorof. and *Dicoccoides* Flaksb. [16, 17, 19–22]. Thus, analysis of tetraploid wheats *T. timopheevii* (Zhuk.) Zhuk. and *T. araraticum* Jakubz. revealed one dominant allele of the *VRN-A1* gene (*Vrn-A1f*) comprising two deletions in the promoter region, and the only dominant allele of *VRN-G1* gene (*Vrn-G1a*) with the insertion in the promoter region [16]. Ten various dominant alleles of the *VRN-A1* gene which possess different mutations in compare to *vrn-A1* allele were identified in different tetraploid wheat species of section *Dicoccoides* Flaksb. Seven of them possess deletions of variable length in the promoter (*Vrn-A1b* (*Vrn-A1b.1*), *Vrn-A1b.2*, *Vrn-A1b.5*, *Vrn-A1b.6, Vrn-A1e*, *Vrn-A1f* and *Vrn-A1d*), one (*Vrn-A1i*) has nucleotide substitution and one (*Vrn-A1a* (*Vrn-A1a.3*)) has a foldback element insertion in the promoter region [16, 17, 19, 20]. The first intron sequence of the *VRN-A1* gene from *T. durum* Desf. cultivar Lebsock was found to contain a large deletion identical to *T. durum* ‘Langdon’ [21, 22]. Further analysis allowed to identify this allele in accessions of *T. turgidum* L., *T. carthlicum* Nevski, *T. polonicum* L., T. *dicoccoides* (Körn. ex Aschers. et Graebn.) Schweinf. and *T. durum* Desf. [17, 19]. Novel allelic variant *VRN-A1f-like* identified by Ivaničová et al. [23] possess mutations in both promoter and first intron regions of *VRN-A1*, while only mutations within first intron is the reason of the spring growth habit of *T. militinae* accession.

In tetraploid wheats of the section *Dicoccoides* four dominant alleles of *VRN-B1* for which the variability is characterized by the mutations in the promoter region (insertion of repeated elements or short deletions) [16, 19, 21]. The *Vrn-B1a* is the only dominant allele with the large deletion in the first intron which was identified in the *Dicoccoides* accessions [19, 22]. The only dominant allele *Vrn-G1a* from section *Timopheevii* is characterized by foldback element insertion in the promoter region [16].

For a long time *vrn-A1* was the only recessive allele identified in hexaploid wheat, but recently additional allele *vrn-A1b.3* was identified in *T. vavilovii* (Thum.) Jakubz. and *T. spelta* L [19]. Seven dominant alleles of the *VRN-A1* gene, *Vrn-A1a.1*, *Vrn-A1a.2*, *Vrn-A1b (Vrn-A1b.1)*, *Vrn-A1b.2*, *Vrn-A1b.6*, *Vrn-A1c* and *Vrn-A1f* were found in hexaploid wheat [16, 19, 20, 22, 23]. The majority of the spring cultivars carry a *Vrn-A1a.1* allele that has a miniature inverted-repeat transposable element (MITE) insertion and duplication in the promoter region [19]. *Vrn-A1b* allelic variants and *Vrn-A1f* allele have mutations and deletions of variable lengths in the promoter region, whereas *Vrn-A1c* has a deletion in the first intron in comparison to the recessive *vrn-A1* allele [16, 19, 20, 22, 23]. Most of the dominant alleles of *VRN-B1* and *VRN-D1* genes possess deletions in the first intron (*Vrn-B1a*, *Vrn-B1b*, *Vrn-B1c* and *Vrn-D1a*) [16, 19, 22, 24–27]. Several recently identified alleles (*Vrn-B1ins, Vrn-D1c, Vrn-D1s*) are characterized by different insertions within the promoter region [19, 28, 29]. The *Vrn*-*D1b* is characterized by the deletion in intron 1 identical to *Vrn*-*D1a* allele and a single nucleotide mutation at promoter and is associated with facultative growth habit [30].

The evolution of spring cultivars of wheats from winter ancestors is a key event in the post-domestication spread of wheat [1]. However, studies of the major vernalization gene *VRN1* are mostly limited to the analysis of di- and hexaploid wheat species. In the present study we investigate the growth habit and variability of promoter and first intron regions of *VRN1* genes in accessions of twelve tetraploid wheat species of sections *Dicoccoides* and *Timopheevii*.

## Methods

### Plant material

Accessions of 12 tetraploid wheat species were obtained from the following gene banks: N.I. Vavilov Institute of Plant Genetic Resources (VIR, Russian Federation), The Federal Research Center Institute of Cytology and Genetics SB RAS (Russian Federation), the National Small Grains Collection (NSGC, USA), International Center for Agricultural Research in the Dry Area (ICARDA, Syria), Kyoto University (Japan). Place of origin, specimen voucher and growth habit of each accession are presented in Additional file 1: Table S1.

### Greenhouse experiments

The growth habit of tetraploid wheat species was evaluated by growing in the greenhouse at 20–25 °C under a long photoperiod (18 h light) without vernalization treatment. F_1_ hybrids of tetraploid near-isogenic line *T. dicoccum* Black Spring *VRN-B1* Emmer (i: BS2E) and *T. dicoccum* (Schrank) Schuebl. cv. Black Winter Emmer (BWE) were used as controls. In i: BS2E, spring growth habit is determined by the dominant allele of the *Vrn-B1* gene [31]. *T. dicoccum* cv. BWE has a winter growth habit. F_1_ hybrids of NIL BS2E with *T. dicoccum* cv. BWE have the genotype *Vrn-B1*/*vrn-B1* and represent the latest maturing spring form at the border between spring vs. winter phenotypes, according to Pugsley [32] and Goncharov [33]. Accessions that headed before F_1_ hybrids were classified as spring, whereas accessions that remained in the vegetative phase were classified as winter. A detailed procedure is described in Goncharov [33].

To determine days to heading of spring accessions ten plants of each accession were grown in the greenhouse at 20–25 °C under a long photoperiod (18 h light) without vernalization treatment. Mean number of days to heading (χ ± *s*) and mean error (*s*) were estimated using standard Microsoft office software.

The number of dominant *VRN* genes in tetraploid wheat was identified based on the segregations in the F_2_ generations. F_1_ hybrids between six accessions of tetraploid wheats and three tester lines, winter accessions *T. dicoccum* cv. BWE, *T. dicoccum* Black Spring *VRN-A1* Emmer (i: BS1E) and i: BS2E, were produced by emasculation of mother’s plant spikes and pollination with flowering father plant spikes using twirl-method. *T. dicoccum* cv. BWE has recessive *vrn-A1* and *vrn-B1* alleles while two near-isogenic test lines are characterized with the only specific dominant allele: *Vrn-A1* in i: BS1E and *Vrn-B1* of i: BS2E [32]. The segregation into spring versus winter forms for each cross was identified and compared with the expected segregation ratio using the Pierson chi-square test.

### Total DNA isolation, PCR amplification, cloning and sequencing

Total DNA was isolated from 100 mg of leaves using the DNeasy Plant Mini Kit (QIAGEN) according to the manufacturer’s protocol. A set of primers were used to amplify the promoter and first intron sequences of *VRN-A1*, *VRN-B1* and *VRN-G1* genes (Additional file 1: Table S2).

Polymerase chain reactions (PCR) were performed in a 20 μl volume with 10 mM Tris–HCl (pH 8.9), 1 mM (NH_4_)_2_SO_4_, 4 mM MgCl_2_, 200 μM of each dNTP, 0.5 μM of each primer, 1 unit of Taq DNA polymerase and 0.1 μg of genomic DNA. The PCR program included an initial denaturation step for 3 min at 94 °C and 33 cycles of amplification consisting of 30 s denaturation at 94 °C, 40 s annealing at 52 °C, and 1 min extension at 72 °C. PCR products were separated by agarose gel electrophoresis and purified using a QIAquick Gel Extraction Kit (QIAGEN). Purified fragments were cloned into a pGEM®-T Easy vector using a pGEM-T Easy kit (Promega) and amplified with M13 primers (M13F = 5′-GTTTTCCCAGTCACGAC-3′, M13R = 5′-AGCGGATAACAATTTCACACAGGA-3′). Sequencing reactions were performed with 200 ng of the product and ABI BigDye Terminator Kit on an ABI 3130XL Genetic Analyser (Applied Biosystems) in SB RAS Genomics Core Facility (http://www.niboch.nsc.ru/doku.php/corefacility). In total 10 clones were sequenced for each target region of all wheat accessions with spring growth habit.

The sequences of the promoter and first intron region of *VRN-A1*, *VRN-B1* and *VRN-G1* genes were deposited in GenBank (accession numbers are given in Tables 1 and 2).

**Table 1:** Studied spring accessions of tetraploid wheats, their heading time and *VRN-A1* gene variability

№	Species and cultivar^a^	Accession/specimen voucher	Sample location (country, site)	Days to heading, χ ± *s*	*VRN-A1* promotor allele	*VRN-A1* intron 1 (intact/with Langdon or IL369 deletion)^b^
Section *Dicoccoides* Flaksb.						
1	*Triticum aethiopicum* Jakubz.	K-18999	Ethiopia, Harari	34.7 ± 0.9	*vrn-A1* KP063952	intact KP063935
2	*T. aethiopicum* Jakubz.	K-19301	Ethiopia, Oromia	43.1 ± 1.8	*vrn-A1* KP063953	*Vrn-A1c* KP063936
3	*T. aethiopicum* Jakubz.	K-19398	Ethiopia, Oromia	37.3 ± 0.0	*vrn-A1* KP063954	*Vrn-A1c* KP063937
4	*T. aethiopicum* Jakubz.	K-19553	Ethiopia, Oromia	36.7 ± 1.8	*vrn-A1* KP063955	*Vrn-A1c* KP063938
5	*T. aethiopicum* Jakubz. cv. Gukur-sindi	K-19253	Ethiopia, Oromia	40.2 ± 2.9	*vrn-A1* KP063956	*Vrn-A1c* KP063939
6	*T. aethiopicum* Jakubz. cv. Gerei	K-19650	Eritrea, Asmara, Ufficio agrario	35.0 ± 0.0	*vrn-A1* KP063957	*Vrn-A1c* KP063940
7	*T. aethiopicum* Jakubz.	K-19059	Ethiopia, Oromia	38.0 ± 0.1	*vrn-A1* KP063958	intact KP063941
8	*T. aethiopicum* Jakubz.	K-43766	Ethiopia, province Shewa, neighborhood of Addis Ababa	34.5 ± 3.0	*vrn-A1* KP063959	*Vrn-A1c*
9	*T. aethiopicum* Jakubz.	St56	Ethiopia	39.4 ± 1.5	*vrn-A1* KP063960	*Vrn-A1c*
10	*T. carthlicum* Nevski	K-7106	Georgia	38.7 ± 1.7	*Vrn-A1e* KP063961	intact
11	*T. dicoccoides* (Körn. ex Aschers. et Graebn.) Schweinf.	PI 467027	Israel, Tabigha	50.9 ± 4.9	*Vrn-A1d* KP063962, *Vrn-A1f* KP063963	intact KP063942
12	*T. dicoccoides* (Körn. ex Aschers. et Graebn.) Schweinf.	PI 467014	Israel, Tabigha	49.0 ± 0.0	*Vrn-A1f* KP063964, *Vrn-A1d* KP063965	intact KP063943
13	*T. dicoccoides* (Körn. ex Aschers. et Graebn.) Schweinf.	PI 467019	Israel, Tabigha	59.0 ± 2.8	*Vrn-A1f* KP063967, *Vrn-A1d* KP063966	intact KP063944
14	*T. dicoccoides* (Körn. ex Aschers. et Graebn.) Schweinf.	PI 428105	Israel, region between Rosh Pinna and Safad	35.5 ± 2.6	*Vrn-A1f* KP063968, *vrn-A1b.4* KP063969	intact KP063945
15	*T. dicoccoides* (Körn. ex Aschers. et Graebn.) Schweinf.	PI 352324	Lebanon, Anti-Lebanon region	30.0 ± 0.0	*Vrn-A1f* KP063971, *Vrn-A1b.7* KP063970	intact
16	*T. dicoccoides* (Körn. ex Aschers. et Graebn.) Schweinf.	PI 352328	Germany, Gatersleben	34.8 ± 3.8	*Vrn-A1f* KP063972, *vrn-A1b.4* KP063973	intact KP063946
17	*T. dicoccoides* (Körn. ex Aschers. et Graebn.) Schweinf.	IG 46225	Turkey, Siverek	78.5 ± 2.5	*vrn-A1* KP063974	intact
18	*T. dicoccoides* (Körn. ex Aschers. et Graebn.) Schweinf.	IG 46223	Turkey, Siverek	54.7 ± 4.3	*Vrn-A1d* KP063975	intact
19	*T. dicoccoides* (Körn. ex Aschers. et Graebn.) Schweinf.	K-62328	Israel, between Migdal and Rosh Pinna	49.8 ± 1.8	*vrn-A1* KP063976	*Vrn-A1c*
20	*T. dicoccoides* (Körn. ex Aschers. et Graebn.) Schweinf.	K-26118	Israel	49.6 ± 1.4	*Vrn-A1d* KP063977	intact
21	*T. dicoccoides* (Körn. ex Aschers. et Graebn.) Schweinf.	ICG №13	Israel, Yehudiyya	59.0 ± 1.0	*Vrn-A1d* KP063980	intact
22	*T. dicoccoides* (Körn. ex Aschers. et Graebn.) Schweinf.	ICG №15	Israel, Yehudiyya	63.7 ± 2.6	*Vrn-A1d* KP063981	intact
23	*T. dicoccoides* (Körn. ex Aschers. et Graebn.) Schweinf.	ICG №18	Israel, Yehudiyya	60.0 ± 3.0	*Vrn-A1d* KP063982	intact
24	*T. dicoccoides* (Körn. ex Aschers. et Graebn.) Schweinf.	ICG №19	Israel, Yehudiyya	49.0 ± 0.2	*Vrn-A1d* KP063983	intact
25	*T. dicoccoides* (Körn. ex Aschers. et Graebn.) Schweinf.	ICG №2	Israel, Arbel	59.0 ± 0.1	*Vrn-A1d* KP063984	intact
26	*T. dicoccoides* (Körn. ex Aschers. et Graebn.) Schweinf.	ICG №23	Israel, Golan Heights	58.9 ± 2.1	*Vrn-A1d* KP063985	intact
27	*T. dicoccoides* (Körn. ex Aschers. et Graebn.) Schweinf.	ICG №24	Israel, Golan Heights	49.7 ± 1.5	*Vrn-A1d* KP063986	intact
28	*T. dicoccoides* (Körn. ex Aschers. et Graebn.) Schweinf.	ICG №27	Israel, Bet-Oren	49.6 ± 1.4	*Vrn-A1d* KP063987	intact
29	*T. dicoccoides* (Körn. ex Aschers. et Graebn.) Schweinf.	ICG №125	Israel	54.2 ± 4.1	*Vrn-A1d* KP063988	intact
30	*T. dicoccoides* (Körn. ex Aschers. et Graebn.) Schweinf.	K-15900	Israel, Migdal	51.3 ± 3.7	*Vrn-A1a.3* KP260494	intact
31	*T. dicoccoides* (Körn. ex Aschers. et Graebn.) Schweinf.	IG 346783	Origin is unknown	50.0 ± 0.2	*Vrn-A1b.2* KP260496, KP260497	intact
32	*T. dicoccum* (Schrank) Schuebl. cv. Krausei	K-20749	Germany	52.0 ± 2.0	*vrn-A1* KP063989	*Vrn-A1c*
33	*T. dicoccum* (Schrank) Schuebl. cv. Dichter Rotlicher	K-1730	Germany	46.3 ± 0.5	*Vrn-A1b.2* KP063990	*Vrn-A1c*
34	*T. dicoccum* (Schrank) Schuebl.	K-7500	Germany	53.3 ± 1.8	*Vrn-A1b.2* KP063991, KP063992	intact KP063947
35	*T. dicoccum* cv. Bastard Emmer verastelter	K-40306	Germany	47.5 ± 2.5	*vrn-A1* KR055694	*Vrn-A1c* KR055695
36	*T. dicoccum* (Schrank) Schuebl i: BS1E	-	Russia	49.6 ± 1.2	*Vrn-A1a.3* GQ451756 [16]	intact
37	*T. durum* Desf. cv. Langdon	-	USA	40.8 ± 1.6	*vrn-A1* KP063995	*Vrn-A1c* KP063948
38	*T. durum* Desf.	K-17784	Cyprus, Nicosia	39.0 ± 0.0	*vrn-A1* KP063996	*Vrn-A1c*
39	*T. durum* Desf.	K-17787	Cyprus, Morphou	35.0 ± 0.1	*vrn-A1* KP063997	*Vrn-A1c* KP063949
40	*T. durum* Desf. cv. Gaza	K-52989	Israel	35.3 ± 0.8	*vrn-A1* KP063998	*Vrn-A1c* KP063950
41	*T. durum* Desf.	K-13768	Armenia, village Voskresenovka	45.5 ± 2.1	*Vrn-A1b.1* KP063999	intact
42	*T. durum* Desf. cv. Nursit	K-18118	Israel	43.2 ± 0.9	*Vrn-A1b.1* KP064000, KP064001	intact
43	*T. durum* Desf.	IG 85879	Jordania	45.6 ± 1.2	*Vrn-A1b.1* KR055675, KR055676	intact
44	*T. polonicum* L.	K-17893	Israel, Center district	55.1 ± 1.1	*Vrn-A1b.1* KP064003, KP064004	*Vrn-A1c*
45	*T. polonicum* L.	K-19597	Ethiopia, Ismala	42.6 ± 2.4	*Vrn-A1b.6* KP064005	intact
46	*T. polonicum* L.	K-43335	China, Xinjiang Uyghur	NI	*Vrn-A1b.6* KP064006	intact
47	*T. turanicum* Jakubz.	K-31693	Tajikistan	39.8 ± 0.3	*vrn-A1* KR055677	intact
48	*T. turgidum* L.	K-3047	Uzbekistan, Tashkent	43.0 ± 1.2	*Vrn-A1b.6* KP064007	intact
49	*T. turgidum* L.	K-13489	Azerbaijan, Cuban county	45.2 ± 1.6	*Vrn-A1b.6* KP064008	intact
50	*T. turgidum* L. cv. Zafrani	K-11597	Afghanistan, village near Herat	32.4 ± 3.6	*vrn-A1* KP064009	*Vrn-A1c*
51	*T. turgidum* L. cv. Maiorka	K-16156	Algeria, neighborhood of Algiers	40.0 ± 0.2	*vrn-A1* KR055679	*Vrn-A1c* KR055680
52	*T. turgidum* L. cv. Blancal	K-20416	Spain, Valencia	NI	*vrn-A1* KR055697	intact KR055698
Section *Timopheevii* A.Filat. et Dorof.						
53	*T. araraticum* Jakubz.	K-58667	Armenia, neighborhood of the village Geghadir	50.9 ± 4.1	*Vrn-A1f* KP064011, *Vrn-A1b.7* KP064012	intact KP063951
54	*T. araraticum* Jakubz.	K-30234	Azerbaijan, Naxcivan	42.4 ± 2.1	*Vrn-A1f* KR055683, KR055684	intact
55	*T. timopheevii* (Zhuk.) Zhuk. Zanduri population	K-38555	Georgia, village Labechina	37.0 ± 3.5	*Vrn-A1f* KR055686	intact
56	*T. timopheevii* (Zhuk.) Zhuk.	K-29540	Georgia	36.4 ± 4.4	*Vrn-A1f* KR055688	intact
57	*T. timopheevii* (Zhuk.) Zhuk.	KU107-1	Georgia	35.4 ± 4.3	*Vrn-A1f* KP064010	intact

^a^species names are given according to Goncharov [41] and Dorofeev et al. [42]

^b^Langdon deletion = first intron sequence with deletion identified in *T. aestivum* NIL Triple Dirk C; IL369 deletion = first intron sequence with deletion identified in hexaploid Afghanistan landrace IL369; intact = first intron sequence without Langdon and IL369 deletion

**Table 2:** *VRN-B1* and *VRN-G1* genes variability in spring accessions of tetraploid wheats used in the study

№	Species and cultivar^a^	Accession/specimen voucher	Sample location (country, site)	Days to heading, χ ± s	*VRN-B1/VRN-G1* promotor allele	*VRN-B1/VRN-G1* intron allele (intact/with TDB deletion) ^b^
Section *Dicoccoides* Flaksb.						
1	*T. aethiopicum* Jakubz.	K-18999	Ethiopia, Harari	34.7 ± 0.9	*vrn-B1* KP063919	*Vrn-B1a* KP063931
2	*T. aethiopicum* Jakubz.	K-19059	Ethiopia, Oromia	38.0 ± 0.1	*vrn-B1* KP063930	intact
3	*T. dicoccoides* (Körn. ex Aschers. et Graebn.) Schweinf.	IG 46225	Turkey, Siverek	78.5 ± 2.5	*vrn-B1* KP063925, *Vrn-B1dic* KP063926	intact KP063932
4	*T. turanicum* Jakubz.	K-31693	Tajikistan	39.8 ± 0.3	*vrn-B1* KR055678	intact
5	*T. turgidum* L. cv. Blancal	K-20416	Spain, Valencia	NI	*vrn-B1* KR055699	intact
Section *Timopheevii *A.Filat. et Dorof.						
6	*T. araraticum* Jakubz.	K-58667	Armenia, neighborhood of the village Geghadir	50.9 ± 4.1	*vrn-G1* KR055682	intact
7	*T. araraticum* Jakubz.	K-30234	Azerbaijan, Naxcivan	42.4 ± 2.1	*Vrn-G1a* KR055685	intact
8	*T. timopheevii* (Zhuk.) Zhuk. Zanduri population	K-38555	Georgia, village Labechina	37.0 ± 3.5	*Vrn-G1a* KR055687	intact
9	*T. timopheevii* (Zhuk.) Zhuk.	K-29540	Georgia	36.4 ± 4.4	*Vrn-G1a* KR055689	intact
10	*T. timopheevii* (Zhuk.) Zhuk.	KU107-1	Georgia	35.4 ± 4.3	*Vrn-G1a* KR055690	intact

^a^species names are given according to Goncharov [41] and Dorofeev et al. [42]

^b^TDB deletion = first intron sequence with deletion identified in *T. aestivum* NIL Triple Dirk B; intact = first intron sequence without TDB deletion

### Sequence analyses

Nucleotide sequence alignments were performed using Vector NTI AdvanceTM version 10.0 program and improved with the MUSCLE algorithm in UGENE software (http://ugene.unipro.ru/) [34, 35].

## Results

### Growth habit of tetraploid wheat species

To analyze the variability of the promoter and first intron regions of *VRN-A1*, *VRN-B1* and *VRN-G1* genes we chose a number of tetraploid wheat accessions, covering all species from both *Timopheevii* and *Dicoccoides* sections (Additional file 1: Table S1).

The growth habit of 228 accessions of tetraploid wheat species was checked by comparison to F_1_ hybrids of i: BS2E and winter *T. dicoccum* cv. BWE (see Methods section). In total, 57 accessions of 10 tetraploid wheat species headed before the hybrids and thus revealed a spring growth habit (Table 1; Additional file 1: Table S1). For each of 57 spring accessions 10 plants were grown under glasshouse conditions and days to heading were recorded (Table 1). 171 accessions of *T. dicoccoides* (Körn. ex Aschers. et Graebn.) Schweinf., *T. ispahanicum* Heslot and *T. karamyschevii* Nevski did not produce shoots without vernalization, thus confirming a winter growth habit (Additional file 1: Table S1).

### *VRN-A1* promoter region variability

**Fig. 1:**
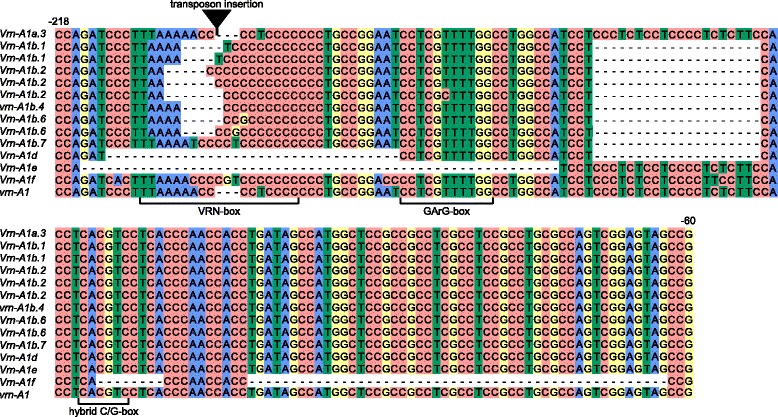
Alignment of *VRN-A1* gene promoter sequences identified from tetraploid wheats in the study. Transposon insertion is indicated by black triangle. Numbers of the nucleotides upstream from the start codon are given in accordance with the sequence *vrn-A1* (GenBank Ac.No GQ451819). Predicted regulatory regions are marked according to Golovnina et al. [16] and Muterko et al. [19]

Mutations within the promoter region appear to be responsible for the major differences between dominant and recessive alleles of the *VRN1* gene and the cause of spring growth habit in wheat plants. Here, 57 accessions were screened using PCR amplification with A genome-specific primers designed by Yan et al. [20]. The PCR product of estimated length (~700 bp) was obtained for all species studied. After cloning of PCR products, all clones obtained were sequenced and analyzed. In total, 69 sequences of the *VRN-A1* promoter region were identified. New sequences were aligned together with known recessive and dominant allelic variants of the *VRN-A1* promoter region in di-, tetra- and hexaploid wheat obtained from GenBank. Comparative analyses demonstrated that the most variable zone of the *VRN-A1* promoter is located in the region from –63 to –220 bp (Fig. 1).

Nine known allelic variants of the *VRN-A1* promoter *Vrn-A1a.3*, *Vrn-A1b*, *Vrn-A1b.2*, *Vrn-A1b.6*, *Vrn-A1d*, *Vrn-A1e*, *Vrn-A1f*, *vrn-A1b.4*, and *vrn-A1* were identified. Promoter sequences of *VRN-A1* from 17 accessions of *T. aethiopicum* Jakubz., *T. dicoccoides*, *T. dicoccum*, *T. durum* Desf. and *T. turgidum* L. were identical to the recessive allele *vrn-A1* identified in polyploids and *vrn-A1u* of *T. urartu* (Table 1, Fig. 1). Four accessions *T. aethiopicum* K-19059, *T. turanicum* Jakubz. K-31693, *T. turgidum* cv. Blancal (K-20416) and *T. turgidum* K-11597 contained one nucleotide substitution in the promoter when compared to the *vrn-A1* sequence (GQ451819).

The other 36 accessions had mutations in the promoter region distinguishing them from the recessive allelic variant *vrn-A1*. The promoter region of the *VRN-A1* gene in *T. dicoccoides* K-15900 and i: BS1E was identical to the *Vrn-A1a.3* allele, and had an insertion of 231 bp in length (Table 1, Fig. 1). Five variants of *Vrn-A1b* allele were identified in accessions *T. dicoccum* cv. Dichter Rotlicher (K-1730), *T. durum* K-13768, *T. turgidum* K-3047 and K-13489, *T. polonicum* K-19597 and K-43335. All sequences of *Vrn-A1b* promoter had common mutation which distinguish them from *vrn-A1* sequence but vary in the length and sequences of VRN-box (Table 1, Fig. 1). Sequences of *T. durum* K-13768 and *T. dicoccum* cv. Dichter Rotlicher (K-1730) were identical to previously described variants *Vrn-A1b.1* and *Vrn-A1b.2*, correspondingly. *VNR-A1* promoter sequences of *T. turgidum* K-13489 and K-3047, *T. polonicum* K-19597 and K-43335 were identical to each other and *Vrn-A1b.6* allele identified in tetraploid and hexaploid wheat species [19].

Five accessions of *Dicoccoides* section had two variants of the *Vrn-A1* promoter sequence, both of which corresponded to the variants of *Vrn-A1b* allele (Table 1). Three accessions *T. durum* K-18118 and IG 85879, and *T. polonicum* L. K-17893 possess identical set of promoter sequences: *Vrn-A1b.1* variant identified previously and sequence differed from *Vrn-A1b.1* only by insertion of one nucleotide “C” in C-rich segment of VRN-box (Fig. 1). Two sequences from accessions of *T. dicoccoides* IG346783 and *T. dicoccum* K-7500 represent *Vrn-A1b.2* variant but vary in the length of C-rich segment (Fig. 1).

The *T. timopheevii* KU107-1, K-29540 and K-38555 had 8 and 50 bp deletions as in the *Vrn-A1f* sequence reported in *T. araraticum* (GQ451762) [16] (Table 1, Fig. 1). One accession of *T. araraticum* K-30234 contained two sequences of the *Vrn-A1f* allele which varied in the length of the C-rich segment of VRN-box.

We observed a 54 bp deletion in the promoter region of *T. carthlicum* K-7106 that was identical to the *Vrn-A1e* alleles of *T. durum* (GQ451821) and *T. dicoccum* (AY616463) (Table 1, Fig. 1).

Eleven accessions of *T. dicoccoides* had two different deletions one 20 bp in length between -136 and -157 and another one 32 bp in length between -179 and -212 nucleotides upstream of the start codon and were identical to the *Vrn-A1d* allele [17, 20] (Table 1, Fig. 1).

The most interesting were two groups of accessions which possessed two different variants of *Vrn-A1* promoter sequences. First group of accession include *T. dicoccoides* PI428105, PI352324 and PI352328 and *T. araraticum* K-58667 which are characterize by the presence of *Vrn-A1f* reported in *T. araraticum* (GQ451762) and one of two variants *Vrn-A1b* allele (Table 1). Second promoter sequences identified in *T. dicoccoides* PI428105 and PI352328 were identical to recessive variant *vrn-A1b.4*. In case of the *T. dicoccoides* PI352324 and *T. araraticum* K-58667 s sequences differed from *vrn-A1* by 20 bp deletion located 137 bp upstream of the start codon, “A- > T” replacement and “CCC” insertion within the VRN-box. This variant was designated as *Vrn-A1b.7* (Fig. 1). Second group of accessions include *T. dicoccoides* PI467027, PI467014 and PI467019 possess two sequences of *VRN-A1* promoter: *Vrn-A1d* and *Vrn-A1f* alleles. Schematic representation of all *VRN1* promoter sequences identified in di- and tetraploid species is presented in Additional file 2: Figure S1. Geographical distribution of the *VRN-A1* alleles identified in the studied samples is presented on Additional file 2: Figure S2.

### *VRN-A1* first intron variability

First intron *VRN-A1* gene in tetraploid wheats was analyzed using three primer pairs (Additional file 1: Table S2). The first primer pair (Ex1/C/F and Intr1/A/R3) allowed us to amplify the sequences of the *VRN-A1* first intron which possesses a deletion of a characteristic length previously described for *T. durum* ‘Langdon’ [22]. The short PCR product (~480 bp in length) containing the deletion was obtained for 7 accessions of *T. aethiopicum* (K-19301, K-19398, K-19553, K-19253, K-19650, K-43766 and St56), 1 accession of *T. dicoccoides* (K-62328), 3 accessions of *T. dicoccum* (K-20749, K-1730 and K-40306), 4 accessions of *T. durum* (K-17784, K-17787, K-52989 and cv. Langdon), 1 accession of *T. polonicum* (K-17893) and 2 accessions of *T. turgidum* (cv. Maiorka (K-16156) and cv. Zafrani (K-11597)) (Table 1). These sequences were identical to the *Vrn-A1c* allele of *T. turgidum* ‘Langdon’, possessing a 7222-bp deletion from 391 bp to 7612 bp compared to the *T. aestivum* NIL Triple Dirk C *vrn-A1* allele (AY747600) [22].

No positive results of PCR amplification were obtained with the primers Intr1/A/F2 and Intr1/A/R3. Thus, no sequences of the *VRN-A1* first intron containing the deletion identified in hexaploid Afghanistan landrace IL369 were presented among analyzed species. The last primer pair (Intr1/C/F and Intr1/AB/R) allowed us to amplify the intact sequences of the first intron, producing a product of the expected length (~1000 bp) for 39 tetraploid wheat accessions (Table 1).

### *VRN-B1* and *VRN-G1* promoter region variability

**Fig. 2:**
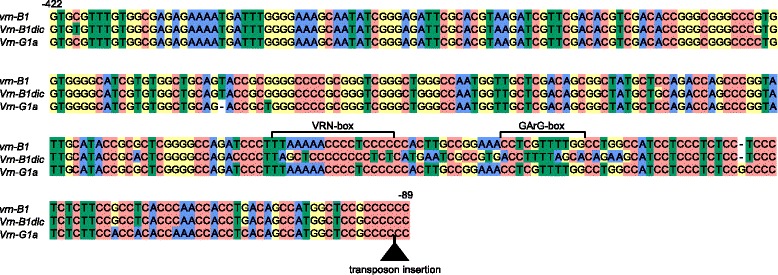
Alignment of *VRN-B1* and *VRN-G1* genes promoters identified from tetraploid wheats in the study. Transposon insertion is indicated by black triangle. Numbers of the nucleotides upstream from the start codon are given in accordance with the sequence *vrn-B1* (GenBank Ac.No AY616453). Predicted regulatory regions are marked according to Golovnina et al. [16] and Muterko et al. [19]

The B genome-specific primers were used to amplify and sequence the promoter region of five accessions of section *Dicoccoides* species*,* with recessive *vrn-A1* promoter sequences and an intact *VRN-A1* first intron. The fragment of the expected length ~ 1200 bp was obtained for all analyzed species. Further comparative analyses demonstrated that almost all clones from accessions of *Dicoccoides* section contained sequences of the intact *vrn-B1* allele (Table 2). The only exception was *T. dicoccoides* IG46225, for which two different clones were identified. The first clone corresponded to the recessive *vrn-B1* alleles identified previously [16, 21, 22]. The second clone differed from the recessive allele by 29 nucleotide substitutions, one deletion and one insertion of a single nucleotide in the region from -220 to -155 bp upstream from the start codon (11 % of dissimilarity) (Table 2, Fig. 2, Additional file 2: Figure S1). The present allelic variant was different from all known B genome alleles of *VRN1* gene and was named *Vrn-B1dic*. No *VRN-B1* promoter sequences containing retrotransposon insertions, previously described by Chu et al. [21], were identified among analyzed accessions.

The *VRN-G1* promoter was analyzed for five accessions of section *Timopheevii* species (Table 2). Sequences of the *VRN-G1* promoter were identified for *T. araraticum* (accession K-30234) and *T. timopheevii* (accessions K-38555, K-29540 and K-29540). The 3′ part of these sequences was identical to the sequence of *Vrn-G1a* allele from *T. timopheevii* K-38555 (GQ451755), while the 5′ end was identified for the first time and differed from *vrn-G1* promoter by two deletions: 8 and 74 bp in length (Fig. 2; Additional file 2: Figure S1). The recessive *vrn-G1* promoter sequences were identified only for accession K-58667 of *T. araraticum*.

### *VRN-B1* and *VRN-G1* first intron variability

First introns of *VRN-B1* and *VRN-G1* genes were analyzed using two primer pairs (see the Methods section). Sequences of the *VRN-B1* first intron possessing the large deletion described for *T. aestivum* NIL Triple Dirk B [22] were amplified with the primers Intr1/B/F and Intr1/B/R3. Positive results of the PCR amplification with this primer pair were obtained for one out of ten studied accessions (Table 2). The sequence of *T. aethiopicum* K-18999 differs from the first intron sequence of *T. aestivum* NIL Triple Dirk B (AY747603) by two nucleotide substitutions. The remaining 9 accessions gave positive results with the second primer pair (Intr1/B/F and Intr1/B/R4), and intact sequences of *VRN-B1* and *VRN-G1* first introns (~1150 bp in length) were amplified (Table 2).

### Genetic control of growth habit in tetraploid species of *Dicoccoides* section

Monogenic or digenic control of growth habit in some spring accessions of tetraploid wheat species of *Dicoccoides* section was analyzed in the genetic experiments. Accessions of tetraploid species were crossed with *T. dicoccum* cv. Black Winter Emmer (BWE) which has recessive *vrn-A1* and *vrn-B1* alleles and two near-isogenic tester lines, which are characterized with specific dominant *Vrn-A1* (i: BS1E) or *Vrn-B1* (i: BS2E) alleles. It was demonstrated that spring growth habit of six accessions of *T. dicoccoides*, *T. dicoccum* and *T. durum* is controlled by a single dominant gene *VRN-A1* (Table 3). F_2_ hybrids of *T. dicoccum* cv. Dichter Rotlicher (K-1730), *T. dicoccum* cv. Bastard Emmer verastelter (K-40306) and *T. durum* cv. Langdon, K-17784 and K-17787 with BWE showed the segregation ration 3 to 1, while their F_2_ hybrids with BS1E showed no segregation and spring growth habit (Table 3). Thus, the results confirmed that the *Vrn-A1c* allele is dominant. For *T. dicoccoides* ICG №23 monogenic control of spring growth habit was shown (Table 3). All F_2_ hybrids of *T. dicoccoides* ICG №23 with BS1E showed spring growth habit which allows us to confirm that newly identified *Vrn-A1d* allele is dominant.

**Table 3:** F_2_ segregation for growth habit in crosses of tetraploid wheat species with three control lines

Accession	Segregation into spring vs. winter forms in the F_2_ generation in crosses with^a^:			Genotype identified by hybrodological method	*VRN-A1* allele identified by molecular methods (promoter/intron1)
	BWE (*vrn-A1 vrn-B1*)	BS1E (*Vrn-A1 vrn-B1*)	BS2E (*vrn-A1 Vrn-B1*)		
*T. dicoccoides* (Körn. ex Aschers. et Graebn.) Schweinf. ICG №23	NA	22:0	95:8^b^	*Vrn-A1 vrn-B1*	*Vrn-A1d*/no Langdon and IL369 deletions
*T. dicoccum* (Schrank) Schuebl. cv. Dichter Rotlicher K-1730	77:22^c^	156:0	NA	*Vrn-A1 vrn-B1*	*Vrn-A1b.2*/*Vrn-A1c*
*T. dicoccum* cv. Bastard Emmer verastelter K-40306	214:93^c^	114:0	NA	*Vrn-A1 vrn-B1*	*vrn-A1*/*Vrn-A1c*
*T. durum* Desf. cv. Langdon	203:40^c^	102:0	NA	*Vrn-A1 vrn-B1*	*vrn-A1*/*Vrn-A1c*
*T. durum* Desf. K-17784	183:63^c^	113:0	NA	*Vrn-A1 vrn-B1*	*vrn-A1*/*Vrn-A1c*
*T. durum* Desf. K-17787	63:14^c^	113:0	NA	*Vrn-A1 vrn-B1*	*vrn-A1*/*Vrn-A1c*

^a^plants thatT before F_1_ hybrids of i: BS2E and BWE were classified as spring, whereas plants that remained in the vegetative phase were classified as winter

^b^digenic control, *χ* ^2^ value for ration is not higher than 3.84

^c^monogenic control, *χ* ^2^ value for ration is not higher than 3.84

A correlation between certain dominant variant of *VRN-A1* genes and number of days to heading as well as a correlation between certain species and number of days to heading were not identified for the studied accessions of tetraploid wheats (Additional file 2: Figure S3, Figure S4).

## Discussion

### Predicted source of the spring growth habit among tetraploid wheat species

Variability in the growth habit (spring vs. winter) of tetraploid wheat has been studied in greenhouse tests and 57 spring accessions were subsequently identified. For four of the species analyzed (*T. polonicum*, *T. carthlicum*, *T. aephiopicum*, and *T. timopheevii*) no winter accessions were identified in the present study nor in a previous study by Goncharov [36]. All studied accessions of *T. ispahanicum* Heslot and *T. karamyschevii* Nevski were found to have a winter growth habit. The following analysis of the *VRN-A1, VRN-B1* and *VRN-G1* genes in accessions with a spring growth habit revealed the presence of different mutations within the promoter or first intron region of those genes.

For 36 of the spring accessions studied, we identified variability within the promoter region of the *VRN-A1* gene (Table 1). *VRN-A1* promoter sequences of these 36 accessions matched one of four different dominant alleles (*Vrn-A1a.3*, *Vrn-A1d*, *Vrn-A1e*, *Vrn-A1f*) or one of four *Vrn-A1b* allele variant (*Vrn-A1b.1, Vrn-A1b.2, Vrn-A1b.6*, *Vrn-A1b.7*). The presence of *Vrn-A1a*, *Vrn-A1b.1, Vrn-A1b.2, Vrn-A1b.6*, *Vrn-A1d*, *Vrn-A1e* or *Vrn-A1f* was previously predicted to be a determinant of the spring growth habit in wheat species [16, 17, 19, 20].

Seven of the 36 spring accessions studied possess two different allelic variants of *VRN-A1*, at least one of which was dominant and could led to the spring growth habit. The presence of two different alleles in one accession could be explained by heterozygosity of the plant material or the variation of copy number of genes due to the duplication of the investigated region or the part of the genome. Presence of two different alleles has not been described for diploid or hexaploid wheat species, but this has been identified in the wild tetraploid species [16, 37].

Allele *vrn-A1* and variants of *Vrn-A1b* were the most frequently occurring, and were identified for 21 and 15 spring tetraploid accessions, respectively (Table 1). *Vrn-A1d* and *Vrn-A1f* were also common and presented in 14 and 11 spring tetraploid accessions, respectively. Dominant inheritance of *Vrn-A1d* was confirmed in the genetic experiments. The remaining two alleles are rare, the *Vrn-A1a* was identified in two accessions while *Vrn-A1e* was found only once (Table 1).

21 accessions of *T. aethiopicum*, *T. dicoccoides*, *T. dicoccum*, *T. durum*, *T. polonicum*, *T. turanicum* and *T. turgidum* contained the recessive allele of the *VRN-A1* promoter. Therefore, its spring growth habit could be explained by other changes in the *VRN1* gene sequences, this may include mutations in the *VRN-B1* promoter regions, as well as in the first intron sequence of both *VRN-A1* and *VRN-B1* genes. 16 out of 21 accessions showed the presence of a large deletion within the *VRN-A1* first intron region (*Vrn-A1c*) and dominant inheritance of this allele was confirmed in the genetic experiments (Table 1, Table 3). Only one of the studied accessions possessed a disruption within the *VRN-B1* promoter sequence. *T. dicoccoides* IG46225 contains two different *VRN-B1* promoters: the first one was identical to the intact sequence of the *vrn-B1* allele, whereas the second one displayed a new allelic variant, named *Vrn-B1dic*. The presence of the *Vrn-B1dic* allele could be the cause of spring growth habit in *T. dicoccoides* IG46225. *Vrn-B1dic* allele is characterized by unexpected high dissimilarity in compare to the *vrn-B1* allele. If we exclude the deletions and insertions cases the other dominant alleles of *VNR1* genes of di- and polyploidy wheats differ from recessive alleles by several SNPs [19, 22, 23, 27, 29]. Alternatively, we could suggest that the *Vrn-B1dic* represent the pseudogene copy originated by duplication within one genome. Previously, the investigation of the bread wheat genome showed the major impact of single gene duplications on the wheat evolution [38]. Moreover the gene duplications followed by gene loss, subfunctionalization or neofunctionalization played significant role in the evolution of MADS-box transcription factors [39]. Investigation of the *Vrn-B1dic* expression is required to proof the hypothesis.

*T. aethiopicum* K-18999 was the only accession for which we identified intact promoters of *VRN-A1* and *VRN-B1* genes, and an intact first intron of the *VRN-A1* gene. A deletion has been found in the first intron of the *VRN-B1* gene. This deletion is a predicted cause of *T. aethiopicum* K-18999’s spring growth habit.

One group of accessions is of particular interest in the investigation of the possible cause of spring growth habit in tetraploid wheat species. *T. dicoccum* cv. Dichter Rotlicher (K-1730) and *T. polonicum* K-17893, both of which posses the dominant allele of the *VRN-A1* promoter, but contain deletions in the first introns of the *VRN-A1* gene. Five accessions of section *Timopheevii* had dominant alleles of both *VRN-A1* and *VRN-G1* promoters. Both variants could contribute to the formation of spring growth habit.

Thus, the predicted source of spring growth habit was determined for 54 of 57 tested accessions, including those described previously as well as the novel disruption identified in the promoter or first intron sequences of *VRN-A1*, *VRN-B1* and/or *VRN-G1* genes. The rest of the accessions, which include *T. aethiopicum* K-19059, *T. turanicum* K-31693 and *T. turgidum* cv. Blancal (K-20416), contained the recessive *VRN-A1* and *VRN-B1* promoter sequences and an intact first intron of both *VRN-A1* and *VRN-B1* genes. A cause for the spring growth habit in these accessions remains unknown.

### Origin of *VRN-A1* promoter variability in tetraploid wheat species

**Fig. 3:**
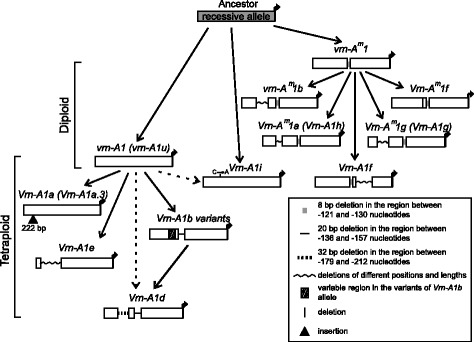
Scheme of the evolution of *VRN-A1* promoter sequences from di- and tetraploid wheat. Arrows indicate the ways of sequences evolution, dotted arrows indicate the alternative ways

**Fig. 4:**
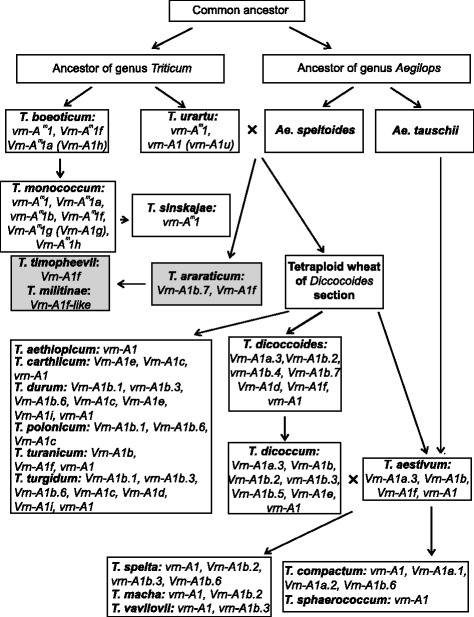
Scheme of *Triticum* and *Aegilops* genera evolution (according to Goncharov [41], with additions). Different alleles of *VRN-A1* gene among wheat species are presented in appropriate boxes next to the species names. Section *Timopheeevii* is presented in grey boxes, while section *Monococcon, Dicoccoides* and *Triticum* are in white boxes

The variability identified in this study is probably the direct cause of the differences between spring and winter growth habit in tetraploid wheat species. To date, 20 different alleles of the *VRN-A1* promoter were identified in the genomes of di- and tetraploid species (Fig. 3; Additional file 2: Figure S1). Three recessive alleles differing by short deletions are presented in diploids, while the remaining four dominant alleles possess different substitutions, deletions and insertions compared to the recessive alleles. Three recessive and eleven dominant are presented among tetraploid accessions (Figs. 3 and 4).

Analysis of the sequences allowed us to suggest the predicted pattern of evolution of *VRN-A1* promoters. The recessive allele *vrn-A1* identified in tetraploid species was inherited from diploids, presumably from *T. urartu* (Figs. 3 and 4).

Dominant alleles *Vrn-A1a* (*Vrn-A1a.3*), *Vrn-A1e*, *Vrn-A1i* and variants of *Vrn-A1b* first appeared in tetraploid species and apparently originated from *vrn-A1* by way of short deletions in the case of *Vrn-A1b* and *Vrn-A1e*, substitution in case of *Vrn-A1i* and insertions for *Vrn-A1a.3* (Fig. 3). The variant of *Vrn-A1b* allele except *Vrn-A1b.7* and *Vrn-A1e* are presented only in tetraploid species of the *Dicoccoides* section, and may originate from the *vrn-A1* allele after sections separation. Allele *Vrn-A1b.7* is presented in both *Dicoccoides* and *Timopheevii* sections and apparently originated in a common tetraploid wheat ancestor before sections separation (Fig. 4). Among tetraploids the distribution of the *Vrn-A1a.3* allele was restricted by *T. dicoccum* and *T. dicoccoides* accessions of the *Dicoccoides* section (Fig. 4).

The dominant *Vrn-A1d* allele is presented in both *Dicoccoides* and *Timopheevii* sections, and may originate from one of the *Vrn-A1b* variants through extension of the deletion. Alternatively, the formation of two deletions in the *vrn-A1* allele could give the *Vrn-A1d* (Fig. 3). Allele *Vrn-A1d* probably originated only once in the ancestor of tetraploid wheat species and evidently was not inherited by hexaploid wheat species (Figs. 3 and 4). Kato et al. [40] predicted that spring accessions of *T. dicoccoides* evolved from a winter forms as an adaptation to warmer conditions. However in the present investigation no correlation between the presence of *Vrn-A1d* allele and particular environmental conditions of collection sites of studied accessions was identified (Table 1).

The most interesting case is the *Vrn-A1f* allele, which in comparison to other dominant alleles of tetraploid wheat, originated from the recessive *vrn-A* ^*m*^ *1* allele of a common ancestor of diploid wheat species (Fig. 3). *Vrn-A1f* allele is on a par with dominant alleles of diploids obtained by deletion in the recessive *vrn-A* ^*m*^ *1* allele promoter. To date, the *Vrn-A1f* allele has been identified in diploids (*T. monococcum*, *T. urartu*, *T. boeoticum*), tetraploids (*Dicoccoides* and *Timopheevii* sections) and hexaploids (*T. aestivum*) (Fig. 4) [16, 17].

## Conclusions

The growth habit was investigated for 228 accessions of 12 tetraploid wheat species. The promoter and first intron regions of *VRN1* genes were analyzed in 57 spring accessions of 10 tetraploid species. Comparative analysis revealed the novel allele of *VRN-A1* (*Vrn-A1b.7*) and *VRN-B1* (*Vrn-B1dic*). *Vrn-A1d* was widely distributed across the accessions of *T. dicoccoides*. In the genetic experiments the dominant mode of inheritance was shown for the *Vrn-A1d* and *Vrn-A1c* alleles. It is assumed that the presence of *Vrn-A1d* allele is associated with the formation of spring growth habit in the 11 accessions of *T. dicoccoides. Vrn-B1dic* is a unique allele characterized by the unexpected high level of promoter sequence dissimilarity in comparison to the *vrn-B1*. This allele was identified in the only accession of *T. dicoccoides* (IG46225) and further investigations are required to determine the role of this allele in the formation of spring growth habit. Novel allelic variants identified in the represent study provide a useful resource for fundamental investigations and could be used in agricultural production to expand the biodiversity of cultivated of wheat species. The summarization of the results regarding to the *VRN1* alleles identified to date in di- and polyploid wheat species allowed us to discuss the evolution of the alleles.

## Additional files

Additional file 1: Table S1. List of tetraploid wheat species used in the study and their growth habit. Species names are given according to Dorofeev et al. [1] and Goncharov [2]. **Table S2.** Set of primers used in the present study. (PDF 444 kb)
Additional file 2: Figure S1. Schematic representation of *VRN-A1* (A), *VRN-B1* and *VRN-G1* (B) promoter region variability identified in di- and tetraploid wheat species. Insertions are indicated by black triangles, insertion lengths are marked under the black triangles. Deletions are indicated by dotted black lines. Hatched rectangles indicate variable region in the variants of *Vrn-A1b* allele. Locations of the deletions and insertions are marked in base pair numbers upstream from the start codon, in accordance with the sequence *vrn-A1u* (GenBank Ac.No GQ451819). * - allele was identified by Golovnina et al. 2010. **Figure S2.** Geographical distribution of different variants of *VRN-A1* gene from accessions of studied tetraploid wheat species. Abbrevations: a - *Vrn-A1a.3*; b - variants of *Vrn-A1b*; d - *Vrn-A1d*; e - *Vrn-A1e*; f - *Vrn-A1f*; u - *vrn-A1* (*vrn-A1u*) allele. Numbers on the right of the graphic symbols match the numbers of studied wheat accessions which possess certain allelic variant. **Figure S3.** Distribution of heading time among studied accessions of ten tetraploid wheat species. Different species are marked by different colors. **Figure S4.** Distribution of heading time among *VRN-A1* alleles identified in studied accessions of ten tetraploid wheat species. Different allelic variants are marked by different colors. (PDF 3302 kb)
