# Supplementary material for: VRN1 genes variability in tetraploid wheat species with a spring growth habit
Source: BMC Plant Biol. 2016 Nov 16;16(Suppl 3):93–106. doi: 10.1186/s12870-016-0924-z (PMC5123248; doi:10.1186/s12870-016-0924-z)
Supplement: Additional file 1: Table S1. — List of tetraploid wheat species used in the study and their growth habit. Species names are given according to Dorofeev et al. [1] and Goncharov [2]. Table S2. Set of primers used in the present study. (PDF 444 kb) [file 12870_2016_924_MOESM1_ESM.pdf]

Additional file 1

Journal: BMC Plant Biology.

Title: *VRN1* genes variability in tetraploid wheat species with a spring growth habit.

Authors: Irina Konopatskaia*, Valeriya Vavilova, Elena Ya. Kondratenko, Alexandr Blinov and Nikolay P. Goncharov.

* - corresponding author: The Federal Research Center Institute of Cytology and Genetics SB RAS, Prospekt Lavrentyeva, 10, Novosibirsk, Russian Federation, 630090; Novosibirsk State University, Pirogova 2, 630090, Novosibirsk, Russian Federation; phone: +7 (383) 363-4969; sormacheva@bionet.nsc.ru

Table S1. List of tetraploid wheat species used in the study and their growth habit. Species names are given according to Dorofeev et al. [1] and Goncharov [2].

№	Species and cultivar	Accession/ specimen voucher	Sample location	Growth habit
**Section *Dicoccoides* Flaksb.**				
1.	*Triticum aethiopicum* Jakubz.	K-18999	Ethiopia	s
2.	*T. aethiopicum* Jakubz.	K-19301	Ethiopia	s
3.	*T. aethiopicum* Jakubz.	K-19398	Ethiopia	s
4.	*T. aethiopicum* Jakubz.	K-19553	Ethiopia	s
5.	*T. aethiopicum* Jakubz. cv. Gukur-sindi	K-19253	Ethiopia	s
6.	*T. aethiopicum* Jakubz.	K-19650	Eritrea	s
7.	*T. aethiopicum* Jakubz.	K-19059	Ethiopia	s
8.	*T. aethiopicum* Jakubz.	K-43766	Ethiopia	s
9.	*T. aethiopicum* Jakubz.	St56	Ethiopia	s
10.	*T. carthlicum* Nevski	K-7106	Georgia	s
11.	*T. dicoccoides* (Körn. ex Aschers. et Graebn.) Schweinf.	PI 352324	Lebanon	s
12.	*T. dicoccoides* (Körn. ex Aschers. et Graebn.) Schweinf.	PI 352328	Germany	s
13.	*T. dicoccoides* (Körn. ex Aschers. et Graebn.) Schweinf.	PI 428105	Israel	s
14.	*T. dicoccoides* (Körn. ex Aschers. et Graebn.) Schweinf.	IG 46225	Turkey	s
15.	*T. dicoccoides* (Körn. ex Aschers. et Graebn.) Schweinf.	IG 46223	Turkey	s
16.	*T. dicoccoides* (Körn. ex Aschers. et Graebn.) Schweinf.	ICG expedition № 1	Turkey	w
17.	*T. dicoccoides* (Körn. ex Aschers. et Graebn.) Schweinf.	ICG expedition № 2	Turkey	w
18.	*T. dicoccoides* (Körn. ex Aschers. et Graebn.) Schweinf.	ICG expedition № 3	Turkey	w
19.	*T. dicoccoides* (Körn. ex Aschers. et Graebn.) Schweinf.	ICG expedition № 4	Turkey	w
20.	*T. dicoccoides* (Körn. ex Aschers. et Graebn.) Schweinf.	ICG expedition № 5	Turkey	w
21.	*T. dicoccoides* (Körn. ex Aschers. et Graebn.) Schweinf.	ICG expedition № 6	Turkey	w
22.	*T. dicoccoides* (Körn. ex Aschers. et Graebn.) Schweinf.	ICG expedition № 7	Turkey	w
23.	*T. dicoccoides* (Körn. ex Aschers. et Graebn.) Schweinf.	ICG expedition № 8	Turkey	w
24.	*T. dicoccoides* (Körn. ex Aschers. et Graebn.) Schweinf.	ICG expedition № 9	Turkey	w
25.	*T. dicoccoides* (Körn. ex Aschers. et Graebn.) Schweinf.	ICG expedition № 10	Turkey	w
26.	*T. dicoccoides* (Körn. ex Aschers. et Graebn.) Schweinf.	ICG expedition № 11	Turkey	w
27.	*T. dicoccoides* (Körn. ex Aschers. et Graebn.) Schweinf.	ICG expedition № 12	Turkey	w
28.	*T. dicoccoides* (Körn. ex Aschers. et Graebn.) Schweinf.	ICG expedition № 13	Turkey	w
29.	*T. dicoccoides* (Körn. ex Aschers. et Graebn.) Schweinf.	ICG expedition № 14	Turkey	w

Table S1, continued.

№	Species and cultivar	Accession/ specimen voucher	Sample location	Growth habit
**Section** ***Dicoccoides*** **Flaksb.**				
30.	*T. dicoccoides* (Körn. ex Aschers. et Graebn.) Schweinf.	ICG expedition № 15	Turkey	w
31.	*T. dicoccoides* (Körn. ex Aschers. et Graebn.) Schweinf.	ICG expedition № 16	Turkey	w
32.	*T. dicoccoides* (Körn. ex Aschers. et Graebn.) Schweinf.	ICG expedition № 17	Turkey	w
33.	*T. dicoccoides* (Körn. ex Aschers. et Graebn.) Schweinf.	ICG expedition № 18	Turkey	w
34.	*T. dicoccoides* (Körn. ex Aschers. et Graebn.) Schweinf.	ICG expedition № 19	Turkey	w
35.	*T. dicoccoides* (Körn. ex Aschers. et Graebn.) Schweinf.	ICG expedition № 20	Turkey	w
36.	*T. dicoccoides* (Körn. ex Aschers. et Graebn.) Schweinf.	ICG expedition № 21	Turkey	w
37.	*T. dicoccoides* (Körn. ex Aschers. et Graebn.) Schweinf.	ICG expedition № 22	Turkey	w
38.	*T. dicoccoides* (Körn. ex Aschers. et Graebn.) Schweinf.	ICG expedition № 23	Turkey	w
39.	*T. dicoccoides* (Körn. ex Aschers. et Graebn.) Schweinf.	ICG expedition № 24	Turkey	w
40.	*T. dicoccoides* (Körn. ex Aschers. et Graebn.) Schweinf.	ICG expedition № 25	Turkey	w
41.	*T. dicoccoides* (Körn. ex Aschers. et Graebn.) Schweinf.	ICG expedition № 26	Turkey	w
42.	*T. dicoccoides* (Körn. ex Aschers. et Graebn.) Schweinf.	ICG expedition № 27	Turkey	w
43.	*T. dicoccoides* (Körn. ex Aschers. et Graebn.) Schweinf.	ICG expedition № 28	Turkey	w
44.	*T. dicoccoides* (Körn. ex Aschers. et Graebn.) Schweinf.	ICG expedition № 29	Turkey	w
45.	*T. dicoccoides* (Körn. ex Aschers. et Graebn.) Schweinf.	ICG expedition № 30	Turkey	w
46.	*T. dicoccoides* (Körn. ex Aschers. et Graebn.) Schweinf.	ICG expedition № 31	Turkey	w
47.	*T. dicoccoides* (Körn. ex Aschers. et Graebn.) Schweinf.	ICG expedition № 32	Turkey	w
48.	*T. dicoccoides* (Körn. ex Aschers. et Graebn.) Schweinf.	ICG expedition № 33	Turkey	w
49.	*T. dicoccoides* (Körn. ex Aschers. et Graebn.) Schweinf.	ICG expedition № 34	Turkey	w
50.	*T. dicoccoides* (Körn. ex Aschers. et Graebn.) Schweinf.	ICG expedition № 36	Turkey	w
51.	*T. dicoccoides* (Körn. ex Aschers. et Graebn.) Schweinf.	ICG expedition № 39	Turkey	w
52.	*T. dicoccoides* (Körn. ex Aschers. et Graebn.) Schweinf.	ICG expedition № 40	Turkey	w
53.	*T. dicoccoides* (Körn. ex Aschers. et Graebn.) Schweinf.	ICG expedition № 42	Turkey	w
54.	*T. dicoccoides* (Körn. ex Aschers. et Graebn.) Schweinf.	ICG expedition № 43	Turkey	w
55.	*T. dicoccoides* (Körn. ex Aschers. et Graebn.) Schweinf.	ICG expedition № 44	Turkey	w
56.	*T. dicoccoides* (Körn. ex Aschers. et Graebn.) Schweinf.	ICG expedition № 45	Turkey	w
57.	*T. dicoccoides* (Körn. ex Aschers. et Graebn.) Schweinf.	ICG expedition № 46	Turkey	w
58.	*T. dicoccoides* (Körn. ex Aschers. et Graebn.) Schweinf.	ICG expedition № 47	Turkey	w
59.	*T. dicoccoides* (Körn. ex Aschers. et Graebn.) Schweinf.	ICG expedition № 49	Turkey	w
60.	*T. dicoccoides* (Körn. ex Aschers. et Graebn.) Schweinf.	ICG expedition № 50	Turkey	w
61.	*T. dicoccoides* (Körn. ex Aschers. et Graebn.) Schweinf.	ICG expedition № 51	Turkey	w
62.	*T. dicoccoides* (Körn. ex Aschers. et Graebn.) Schweinf.	ICG expedition № 52	Turkey	w
63.	*T. dicoccoides* (Körn. ex Aschers. et Graebn.) Schweinf.	ICG expedition № 53	Turkey	w
64.	*T. dicoccoides* (Körn. ex Aschers. et Graebn.) Schweinf.	ICG expedition № 54	Turkey	w
65.	*T. dicoccoides* (Körn. ex Aschers. et Graebn.) Schweinf.	ICG expedition № 55	Turkey	w
66.	*T. dicoccoides* (Körn. ex Aschers. et Graebn.) Schweinf.	ICG expedition № 56	Turkey	w
67.	*T. dicoccoides* (Körn. ex Aschers. et Graebn.) Schweinf.	ICG expedition № 57	Turkey	w
68.	*T. dicoccoides* (Körn. ex Aschers. et Graebn.) Schweinf.	ICG expedition № 101	Turkey	w
69.	*T. dicoccoides* (Körn. ex Aschers. et Graebn.) Schweinf.	ICG expedition № 103	Turkey	w
70.	*T. dicoccoides* (Körn. ex Aschers. et Graebn.) Schweinf.	ICG expedition № 104	Turkey	w
71.	*T. dicoccoides* (Körn. ex Aschers. et Graebn.) Schweinf.	ICG expedition № 105	Turkey	w
72.	*T. dicoccoides* (Körn. ex Aschers. et Graebn.) Schweinf.	ICG expedition № 108	Turkey	w
73.	*T. dicoccoides* (Körn. ex Aschers. et Graebn.) Schweinf.	ICG expedition № 111	Turkey	w
74.	*T. dicoccoides* (Körn. ex Aschers. et Graebn.) Schweinf.	ICG expedition № 113	Turkey	w
75.	*T. dicoccoides* (Körn. ex Aschers. et Graebn.) Schweinf.	ICG expedition № 115	Turkey	w
76.	*T. dicoccoides* (Körn. ex Aschers. et Graebn.) Schweinf.	ICG expedition № 116	Turkey	w
77.	*T. dicoccoides* (Körn. ex Aschers. et Graebn.) Schweinf.	ICG expedition № 120	Turkey	w
78.	*T. dicoccoides* (Körn. ex Aschers. et Graebn.) Schweinf.	ICG expedition № 121	Turkey	w
79.	*T. dicoccoides* (Körn. ex Aschers. et Graebn.) Schweinf.	ICG expedition № 122	Turkey	w

Table S1, continued.

№	Species and cultivar	Accession/ specimen voucher	Sample location	Growth habit
**Section *Dicoccoides* Flaksb.**				
80.	*T. dicoccoides* (Körn. ex Aschers. et Graebn.) Schweinf.	ICG expedition № 123	Turkey	w
81.	*T. dicoccoides* (Körn. ex Aschers. et Graebn.) Schweinf.	ICG expedition № 124	Turkey	w
82.	*T. dicoccoides* (Körn. ex Aschers. et Graebn.) Schweinf.	ICG expedition № 125	Turkey	w
83.	*T. dicoccoides* (Körn. ex Aschers. et Graebn.) Schweinf.	ICG expedition № 127	Turkey	w
84.	*T. dicoccoides* (Körn. ex Aschers. et Graebn.) Schweinf.	ICG expedition № 129	Turkey	w
85.	*T. dicoccoides* (Körn. ex Aschers. et Graebn.) Schweinf.	ICG expedition № 161	Turkey	w
86.	*T. dicoccoides* (Körn. ex Aschers. et Graebn.) Schweinf.	ICG expedition № 176	Turkey	w
87.	*T. dicoccoides* (Körn. ex Aschers. et Graebn.) Schweinf.	ICG expedition № 185	Turkey	w
88.	*T. dicoccoides* (Körn. ex Aschers. et Graebn.) Schweinf.	ICG expedition № 187	Turkey	w
89.	*T. dicoccoides* (Körn. ex Aschers. et Graebn.) Schweinf.	ICG expedition № 188	Turkey	w
90.	*T. dicoccoides* (Körn. ex Aschers. et Graebn.) Schweinf.	ICG expedition № 192	Turkey	w
91.	*T. dicoccoides* (Körn. ex Aschers. et Graebn.) Schweinf.	ICG expedition № k-2	Turkey	w
92.	*T. dicoccoides* (Körn. ex Aschers. et Graebn.) Schweinf.	ICG expedition № 661	Turkey	w
93.	*T. dicoccoides* (Körn. ex Aschers. et Graebn.) Schweinf.	ICG expedition № 1-4	Turkey	w
94.	*T. dicoccoides* (Körn. ex Aschers. et Graebn.) Schweinf.	ICG expedition № 6-4-08	Turkey	w
95.	*T. dicoccoides* (Körn. ex Aschers. et Graebn.) Schweinf.	ICG expedition № 233	Turkey	w
96.	*T. dicoccoides* (Körn. ex Aschers. et Graebn.) Schweinf.	ICG expedition № 244	Turkey	w
97.	*T. dicoccoides* (Körn. ex Aschers. et Graebn.) Schweinf.	ICG expedition № 410	Turkey	w
98.	*T. dicoccoides* (Körn. ex Aschers. et Graebn.) Schweinf.	ICG expedition № 414	Turkey	w
99.	*T. dicoccoides* (Körn. ex Aschers. et Graebn.) Schweinf.	ICG expedition № 422	Turkey	w
100.	*T. dicoccoides* (Körn. ex Aschers. et Graebn.) Schweinf.	ICG expedition № 515	Turkey	w
101.	*T. dicoccoides* (Körn. ex Aschers. et Graebn.) Schweinf.	ICG expedition № 520	Turkey	w
102.	*T. dicoccoides* (Körn. ex Aschers. et Graebn.) Schweinf.	ICG expedition № 522	Turkey	w
103.	*T. dicoccoides* (Körn. ex Aschers. et Graebn.) Schweinf.	ICG expedition № 525	Turkey	w
104.	*T. dicoccoides* (Körn. ex Aschers. et Graebn.) Schweinf.	ICG expedition № 527	Turkey	w
105.	*T. dicoccoides* (Körn. ex Aschers. et Graebn.) Schweinf.	ICG expedition № 537	Turkey	w
106.	*T. dicoccoides* (Körn. ex Aschers. et Graebn.) Schweinf.	ICG expedition № 13-1-88	Turkey	w
107.	*T. dicoccoides* (Körn. ex Aschers. et Graebn.) Schweinf.	IG 46148	Turkey	w
108.	*T. dicoccoides* (Körn. ex Aschers. et Graebn.) Schweinf.	IG 116181	Turkey	w
109.	*T. dicoccoides* (Körn. ex Aschers. et Graebn.) Schweinf.	IG 46191	Turkey	w
110.	*T. dicoccoides* (Körn. ex Aschers. et Graebn.) Schweinf.	IG 46149	Turkey	w
111.	*T. dicoccoides* (Körn. ex Aschers. et Graebn.) Schweinf.	IG 46165	Turkey	w
112.	*T. dicoccoides* (Körn. ex Aschers. et Graebn.) Schweinf.	IG 46167	Turkey	w
113.	*T. dicoccoides* (Körn. ex Aschers. et Graebn.) Schweinf.	IG 46171	Turkey	w
114.	*T. dicoccoides* (Körn. ex Aschers. et Graebn.) Schweinf.	IG 46173	Turkey	w
115.	*T. dicoccoides* (Körn. ex Aschers. et Graebn.) Schweinf.	IG 46191	Turkey	w
116.	*T. dicoccoides* (Körn. ex Aschers. et Graebn.) Schweinf.	IG 46175	Turkey	w
117.	*T. dicoccoides* (Körn. ex Aschers. et Graebn.) Schweinf.	IG 46177	Turkey	w
118.	*T. dicoccoides* (Körn. ex Aschers. et Graebn.) Schweinf.	IG 46180	Turkey	w
119.	*T. dicoccoides* (Körn. ex Aschers. et Graebn.) Schweinf.	IG 46181	Turkey	w
120.	*T. dicoccoides* (Körn. ex Aschers. et Graebn.) Schweinf.	IG 46182	Turkey	w
121.	*T. dicoccoides* (Körn. ex Aschers. et Graebn.) Schweinf.	IG 46183	Turkey	w
122.	*T. dicoccoides* (Körn. ex Aschers. et Graebn.) Schweinf.	IG 46206	Turkey	w
123.	*T. dicoccoides* (Körn. ex Aschers. et Graebn.) Schweinf.	IG 46207	Turkey	w
124.	*T. dicoccoides* (Körn. ex Aschers. et Graebn.) Schweinf.	IG 46210	Turkey	w
125.	*T. dicoccoides* (Körn. ex Aschers. et Graebn.) Schweinf.	IG 46212	Turkey	w
126.	*T. dicoccoides* (Körn. ex Aschers. et Graebn.) Schweinf.	IG 46216	Turkey	w
127.	*T. dicoccoides* (Körn. ex Aschers. et Graebn.) Schweinf.	IG 46218	Turkey	w
128.	*T. dicoccoides* (Körn. ex Aschers. et Graebn.) Schweinf.	IG 46219	Turkey	w
129.	*T. dicoccoides* (Körn. ex Aschers. et Graebn.) Schweinf.	IG 46226	Turkey	w
130.	*T. dicoccoides* (Körn. ex Aschers. et Graebn.) Schweinf.	IG 46233	Turkey	w

Table S1, continued.

№	Species and cultivar	Accession/ specimen voucher	Sample location	Growth habit
**Section *Dicoccoides* Flaksb.**				
131.	*T. dicoccoides* (Körn. ex Aschers. et Graebn.) Schweinf.	IG46236	Turkey	w
132.	*T. dicoccoides* (Körn. ex Aschers. et Graebn.) Schweinf.	IG46239	Turkey	w
133.	*T. dicoccoides* (Körn. ex Aschers. et Graebn.) Schweinf.	IG 46250	Turkey	w
134.	*T. dicoccoides* (Körn. ex Aschers. et Graebn.) Schweinf.	IG 46244	Turkey	w
135.	*T. dicoccoides* (Körn. ex Aschers. et Graebn.) Schweinf.	IG 46252	Turkey	w
136.	*T. dicoccoides* (Körn. ex Aschers. et Graebn.) Schweinf.	IG 46253	Turkey	w
137.	*T. dicoccoides* (Körn. ex Aschers. et Graebn.) Schweinf.	IG 116171	Turkey	w
138.	*T. dicoccoides* (Körn. ex Aschers. et Graebn.) Schweinf.	IG 116172	Turkey	w
139.	*T. dicoccoides* (Körn. ex Aschers. et Graebn.) Schweinf.	IG 116173	Turkey	w
140.	*T. dicoccoides* (Körn. ex Aschers. et Graebn.) Schweinf.	IG 116174	Turkey	w
141.	*T. dicoccoides* (Körn. ex Aschers. et Graebn.) Schweinf.	IG 116175	Turkey	w
142.	*T. dicoccoides* (Körn. ex Aschers. et Graebn.) Schweinf.	IG 116180	Turkey	w
143.	*T. dicoccoides* (Körn. ex Aschers. et Graebn.) Schweinf.	IG 116183	Turkey	w
144.	*T. dicoccoides* (Körn. ex Aschers. et Graebn.) Schweinf.	IG 116184	Turkey	w
145.	*T. dicoccoides* (Körn. ex Aschers. et Graebn.) Schweinf.	IG 116185	Turkey	w
146.	*T. dicoccoides* (Körn. ex Aschers. et Graebn.) Schweinf.	IG 116188	Turkey	w
147.	*T. dicoccoides* (Körn. ex Aschers. et Graebn.) Schweinf.	IG 116178	Turkey	w
148.	*T. dicoccoides* (Körn. ex Aschers. et Graebn.) Schweinf.	IG 116179	Turkey	w
149.	*T. dicoccoides* (Körn. ex Aschers. et Graebn.) Schweinf.	IG 45964	Jordan	w
150.	*T. dicoccoides* (Körn. ex Aschers. et Graebn.) Schweinf.	IG 46486	Jordan	w
151.	*T. dicoccoides* (Körn. ex Aschers. et Graebn.) Schweinf.	IG 46357	Jordan	w
152.	*T. dicoccoides* (Körn. ex Aschers. et Graebn.) Schweinf.	IG 46493	Jordan	w
153.	*T. dicoccoides* (Körn. ex Aschers. et Graebn.) Schweinf.	IG 115811	Jordan	w
154.	*T. dicoccoides* (Körn. ex Aschers. et Graebn.) Schweinf.	PI 470736	Turkey	w
155.	*T. dicoccoides* (Körn. ex Aschers. et Graebn.) Schweinf.	IG 46183	Turkey	w
156.	*T. dicoccoides* (Körn. ex Aschers. et Graebn.) Schweinf.	PI 554584	Turkey	w
157.	*T. dicoccoides* (Körn. ex Aschers. et Graebn.) Schweinf.	PI 560873	Turkey	w
158.	*T. dicoccoides* (Körn. ex Aschers. et Graebn.) Schweinf.	PI 560877	Turkey	w
159.	*T. dicoccoides* (Körn. ex Aschers. et Graebn.) Schweinf.	PI 654334	Turkey	w
160.	*T. dicoccoides* (Körn. ex Aschers. et Graebn.) Schweinf.	PI 654336	Turkey	w
161.	*T. dicoccoides* (Körn. ex Aschers. et Graebn.) Schweinf.	PI 656865	Turkey	w
162.	*T. dicoccoides* (Körn. ex Aschers. et Graebn.) Schweinf.	PI 656868	Turkey	w
163.	*T. dicoccoides* (Körn. ex Aschers. et Graebn.) Schweinf.	PI 656870	Turkey	w
164.	*T. dicoccoides* (Körn. ex Aschers. et Graebn.) Schweinf.	PI 656873	Turkey	w
165.	*T. dicoccoides* (Körn. ex Aschers. et Graebn.) Schweinf.	ICG expedition № 1	Israel	w
166.	*T. dicoccoides* (Körn. ex Aschers. et Graebn.) Schweinf.	ICG expedition № 2	Israel	w
167.	*T. dicoccoides* (Körn. ex Aschers. et Graebn.) Schweinf.	ICG expedition № 3	Israel	w
168.	*T. dicoccoides* (Körn. ex Aschers. et Graebn.) Schweinf.	ICG expedition № 4	Israel	w
169.	*T. dicoccoides* (Körn. ex Aschers. et Graebn.) Schweinf.	ICG expedition № 5	Israel	w
170.	*T. dicoccoides* (Körn. ex Aschers. et Graebn.) Schweinf.	ICG expedition № 6	Israel	w
171.	*T. dicoccoides* (Körn. ex Aschers. et Graebn.) Schweinf.	ICG expedition № 7	Israel	w
172.	*T. dicoccoides* (Körn. ex Aschers. et Graebn.) Schweinf.	ICG expedition № 8	Israel	w
173.	*T. dicoccoides* (Körn. ex Aschers. et Graebn.) Schweinf.	ICG expedition № 9	Israel	w
174.	*T. dicoccoides* (Körn. ex Aschers. et Graebn.) Schweinf.	ICG expedition № 10	Israel	w
175.	*T. dicoccoides* (Körn. ex Aschers. et Graebn.) Schweinf.	ICG expedition № 11	Israel	w
176.	*T. dicoccoides* (Körn. ex Aschers. et Graebn.) Schweinf.	ICG expedition № 12	Israel	w
177.	*T. dicoccoides* (Körn. ex Aschers. et Graebn.) Schweinf.	ICG expedition № 13	Israel	w
178.	*T. dicoccoides* (Körn. ex Aschers. et Graebn.) Schweinf.	ICG expedition № 14	Israel	w
179.	*T. dicoccoides* (Körn. ex Aschers. et Graebn.) Schweinf.	ICG expedition № 15	Israel	w
180.	*T. dicoccoides* (Körn. ex Aschers. et Graebn.) Schweinf.	PI 467027	Israel	s
181.	*T. dicoccoides* (Körn. ex Aschers. et Graebn.) Schweinf.	PI 467014	Israel	s

Table S1, continued.

№	Species and cultivar	Accession/ specimen voucher	Sample location	Growth habit
**Section *Dicoccoides* Flaksb.**				
182	*T. dicoccoides* (Körn. ex Aschers. et Graebn.) Schweinf.	PI 467019	Israel	s
183	*T. dicoccoides* (Körn. ex Aschers. et Graebn.) Schweinf.	K-62328	Israel	s
184	*T. dicoccoides* (Körn. ex Aschers. et Graebn.) Schweinf.	K-26118	Israel	s
185	*T. dicoccoides* (Körn. ex Aschers. et Graebn.) Schweinf.	ICG №13	Israel	s
186	*T. dicoccoides* (Körn. ex Aschers. et Graebn.) Schweinf.	ICG №15	Israel	s
187	*T. dicoccoides* (Körn. ex Aschers. et Graebn.) Schweinf.	ICG №18	Israel	s
188	*T. dicoccoides* (Körn. ex Aschers. et Graebn.) Schweinf.	ICG №19	Israel	s
189	*T. dicoccoides* (Körn. ex Aschers. et Graebn.) Schweinf.	ICG №2	Israel	s
190	*T. dicoccoides* (Körn. ex Aschers. et Graebn.) Schweinf.	ICG №23	Israel	s
191	*T. dicoccoides* (Körn. ex Aschers. et Graebn.) Schweinf.	ICG №24	Israel	s
192	*T. dicoccoides* (Körn. ex Aschers. et Graebn.) Schweinf.	ICG №27	Israel	s
193	*T. dicoccoides* (Körn. ex Aschers. et Graebn.) Schweinf.	ICG №125	Israel	s
194	*T. dicoccoides* (Körn. ex Aschers. et Graebn.) Schweinf.	K-15900	Israel	s
195	*T. dicoccoides* (Körn. ex Aschers. et Graebn.) Schweinf.	IG 346783	Azerbaijan	s
196	*T. dicoccum* (Schrank) Schuebl. cv. Krausei	K-20749	Germany	s
197	*T. dicoccum* (Schrank) Schuebl. cv. Dichter Rotlicher	K-1730	Germany	s
198	*T. dicoccum* (Schrank) Schuebl.	K-7500	Germany	s
199	*T. dicoccum* cv. Bastard Emmer verastelter	K-40306	Germany	s
200	*T. dicoccum* (Schrank) Schuebl i: BS1E	-	Russia	s
201	*T. durum* Desf. cv. Langdon	-	USA	s
202	*T. durum* Desf.	K-17784	Cyprus	s
203	*T. durum* Desf.	K-17787	Cyprus	s
204	*T. durum* Desf. cv. Gaza	K-52989	Israel	s
205	*T. durum* Desf.	K-13768	Armenia	s
206	*T. durum* Desf. cv. Nursit	K-18118	Israel	s
207	*T. durum* Desf.	IG 85879	Jordania	s
208	*T. ispahanicum* Heslot	KU 145	Iran	w
209	*T. ispahanicum* Heslot	K-51762	Iran	w
210	*T. ispahanicum* Heslot	K-43064	Iran	w
211	*T. karamyschevii* Nevski	K-28162	Georgia	w
212	*T. karamyschevii* Nevski	K-28205	Georgia	w
213	*T. karamyschevii* Nevski	K-38549	Georgia	w
214	*T. karamyschevii* Nevski	KU 190-2	Georgia	w
215	*T. polonicum* L.	K-17893	Israel	s
216	*T. polonicum* L.	K-19597	Ethiopia	s
217	*T. polonicum* L.	K-43335	China	s
218	*T. turanicum*	K-31693	Tajikistan	s
219	*T. turgidum* L.	K-3047	Uzbekistan	s
220	*T. turgidum* L.	K-13489	Azerbaijan	s
221	*T. turgidum* L. cv. Zafrani	K-11597	Afghanistan	s
222	*T. turgidum* L. cv. Maiorka	K-16156	Algeria	s
223	*T. turgidum* L. cv. Blancal	K-20416	Spain	s
**Section *Timopheevii* A.Filet. et Dorof.**				
224	*T. araraticum* Jakubz.	K-58667	Armenia	s
225	*T. araraticum* Jakubz.	K-30234	Azerbaijan	s
226	*T. timopheevii* (Zhuk.) Zhuk. Zanduri population	K-38555	Georgia	s
227	*T. timopheevii* (Zhuk.) Zhuk.	K-29540	Georgia	s
228	*T. timopheevii* (Zhuk.) Zhuk.	KU107-1	Georgia	s

Table S2. Set of primers used in the present study.

Primers name	Primers sequence	Target sequence	Reference
VRN1AF<br>VRN1-INT1R	5'-GAAAGGAAAAATTCTGCTCG-3'<br>5'-GCAGGAAATCGAAATCGAAG-3'	*VRN-A1* promoter	[3]
Intr1/C/F<br>Intr1/AB/R	5'-GCACTCCTAACCCACTAACC-3'<br>5'-TCATCCATCATCAAGGCAAA-3'	*VRN-A1* intron 1	[4]
Ex1/C/F<br>Intr1/A/R3	5'-GTTCTCCACCGAGTCATGGT-3'<br>5'-AAGTAAGACAACACGAATGTGAGA-3'	*VRN-A1* intron 1 with Langdon deletion	[4]
Intr1/A/F2<br>Intr1/A/R3	5'-AGCCTCCACGGTTTGAAAGTAA-3'<br>5'-AAGTAAGACAACACGAATGTGAGA-3'	*VRN-A1* intron 1 with IL369 deletion	[4]
VRN1BF<br>VRN1-INT1R	5'-CAGTACCCCTGCTACCAGTG-3'<br>5'-GCAGGAAATCGAAATCGAAG-3'	*VRN-B1* promoter, *VRN-G1* promoter	[3]
Intr1/B/F<br>Intr1/B/R4	5'-CAAGTGGAACGGTTAGGACA-3'<br>5'-CAAATGAAAAGGAATGAGAGCA-3'	*VRN-B1* and *VRN-G1* intron 1	[4]
Intr1/B/F<br>Intr1/B/R3	5'-CAAGTGGAACGGTTAGGACA-3'<br>5'-TCATGCCAAAAATTGAAGATGA-3'	*VRN-B1* intron 1 with NIL Triple Dirk B deletion	[4]

References

1. Dorofeev VF, Filatenko AA, Migushova FF, Udachin RA, Jakubtsiner MM, Cultivated Flora of the USSR. Vol. 1. Pshenitsa (Wheat). Kolos, Leningrad, 1979 (In Russian).

2. Goncharov NP. Genus *Triticum* L. taxonomy: the present and the future. Plant Syst Evol. 2011;295(1):1-11.

3. Fu D, Szucs P, Yan L, Helguera M, Skinner JS, von Zitzewitz J, Hayes PM, Dubcovsky J. Large deletions within the first intron in *VRN-1* are associated with spring growth habit in barley and wheat. Mol Genet Genomics. 2005;273(1):54-65.

4. Yan L, Helguera M, Kato K, Fukuyama S, Sherman J, Dubcovsky J. Allelic variation at the *VRN-1* promoter region in polyploid wheat. Theor Appl Genet. 2004;109(8):1677-86.
