# Supplementary material for: VRN1 genes variability in tetraploid wheat species with a spring growth habit
Source: BMC Plant Biol. 2016 Nov 16;16(Suppl 3):93–106. doi: 10.1186/s12870-016-0924-z (PMC5123248; doi:10.1186/s12870-016-0924-z)
Supplement: Additional file 2: Figure S1. — Schematic representation of VRN-A1 (A), VRN-B1 and VRN-G1 (B) promoter region variability identified in di- and tetraploid wheat species. Insertions are indicated by black triangles, insertion lengths are marked under the black triangles. Deletions are indicated by dotted black lines. Hatched rectangles indicate variable region in the variants of Vrn-A1b allele. Locations of the deletions and insertions are marked in base pair numbers upstream from the start codon, in accordance with the sequence vrn-A1u (GenBank Ac.No GQ451819). * - allele was identified by Golovnina et al. 2010. Figure S2. Geographical distribution of different variants of VRN-A1 gene from accessions of studied tetraploid wheat species. Abbrevations: a - Vrn-A1a.3; b - variants of Vrn-A1b; d - Vrn-A1d; e - Vrn-A1e; f - Vrn-A1f; u - vrn-A1 (vrn-A1u) allele. Numbers on the right of the graphic symbols match the numbers of studied wheat accessions which possess certain allelic variant. Figure S3. Distribution of heading time among studied accessions of ten tetraploid wheat species. Different species are marked by different colors. Figure S4. Distribution of heading time among VRN-A1 alleles identified in studied accessions of ten tetraploid wheat species. Different allelic variants are marked by different colors. (PDF 3302 kb) [file 12870_2016_924_MOESM2_ESM.pdf]

**Additional file 2**

**Journal**: BMC Plant Biology.

**Title**: *VRN1* genes variability in tetraploid wheat species with a spring growth habit.

**Authors:** Irina Konopatskaia*, Valeriya Vavilova, Elena Ya. Kondratenko, Alexandr Blinov and Nikolay P. Goncharov.
* - corresponding author: The Federal Research Center Institute of Cytology and Genetics SB RAS, Prospekt Lavrentyeva,10, Novosibirsk, Russian Federation, 630090; phone: +7 (383) 363-4969; sormacheva@bionet.nsc.ru

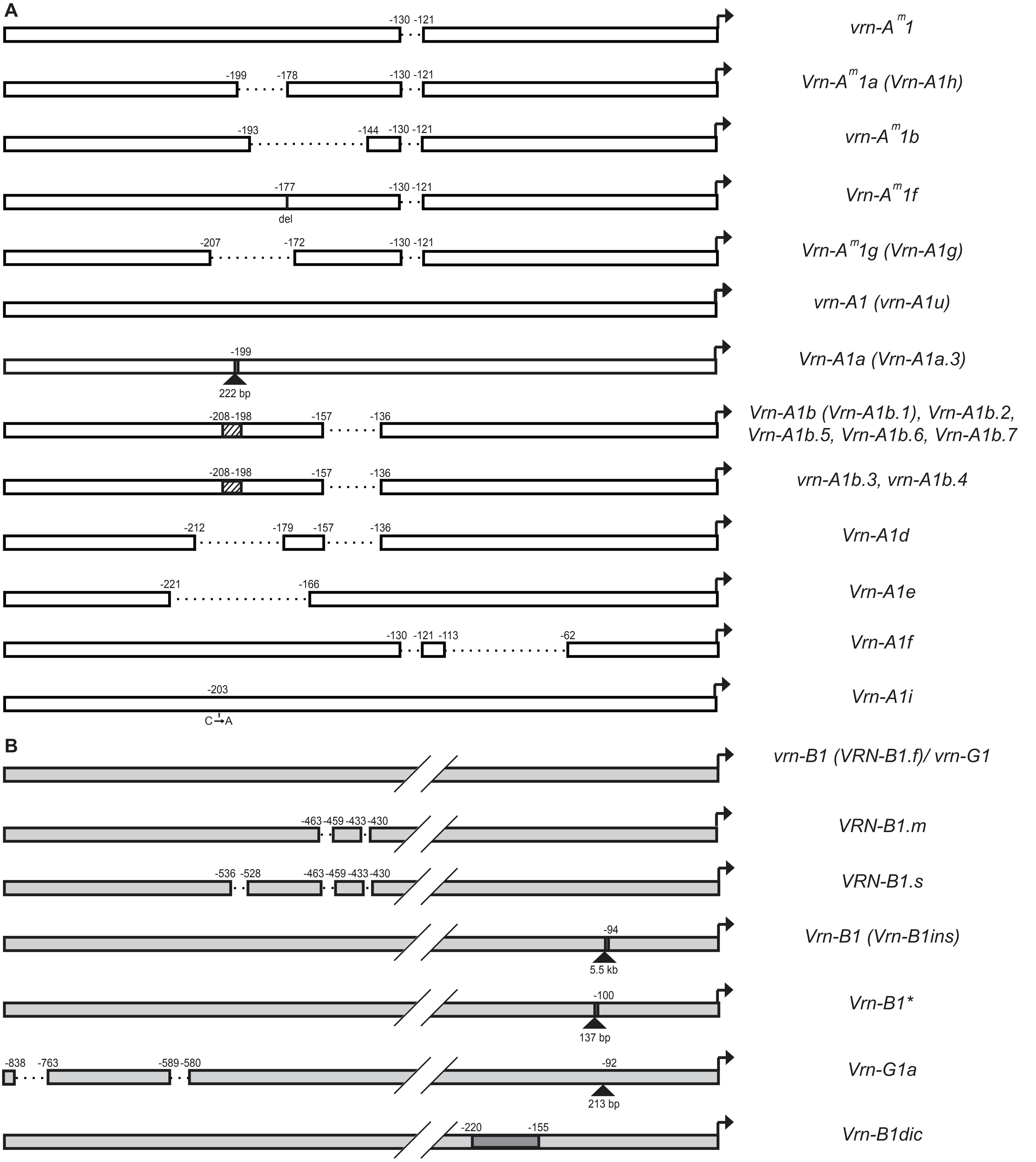


**Figure S1**. Schematic representation of *VRN-A1* (A), *VRN-B1* and *VRN-G1* (B) promoter region variability identified in di- and tetraploid wheat species. Insertions are indicated by black triangles, insertion lengths are marked under the black triangles. Deletions are indicated by dotted black lines. Hatched rectangles indicate variable region in the variants of *Vrn-A1b* allele. Locations of the deletions and insertions are marked in base pair numbers upstream from the start codon, in accordance with the sequence *vrn-A1u* (GenBank Ac.No GQ451819).
* - allele was identified by Golovnina et al. 2010.

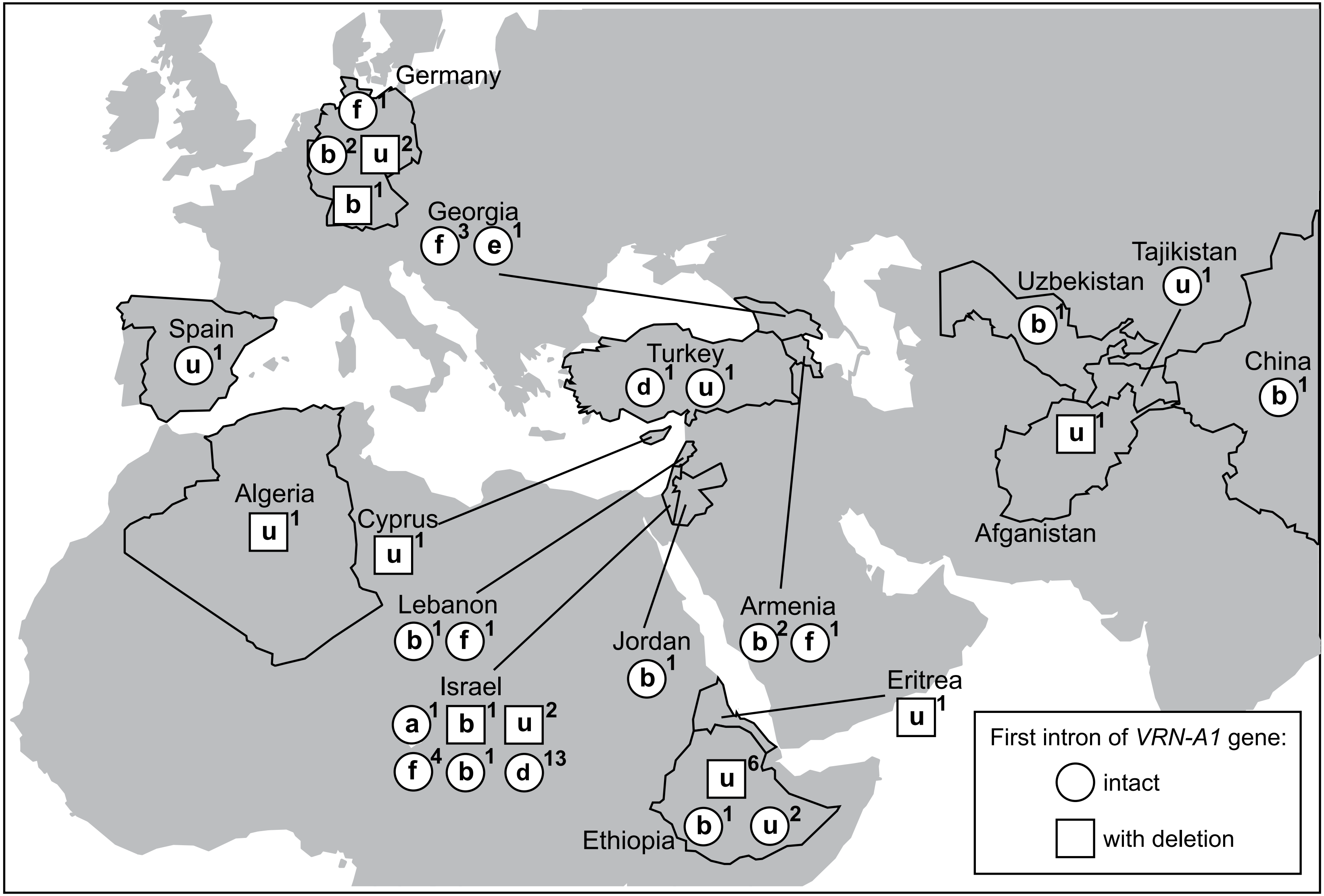


**Figure S2.** Geographical distribution of different variants of *VRN-A1* gene from accessions of studied tetraploid wheats. Abbrevations: a - *Vrn-A1a.3*; b - variants of *Vrn-A1b*; d - *Vrn-A1d*; e - *Vrn-A1e*; f - *Vrn-A1f*; u - *vrn-A1* (*vrn-A1u*) allele. Numbers on the right of the graphic symbols match the numbers of studied wheat accessions which possess certain allelic variant.

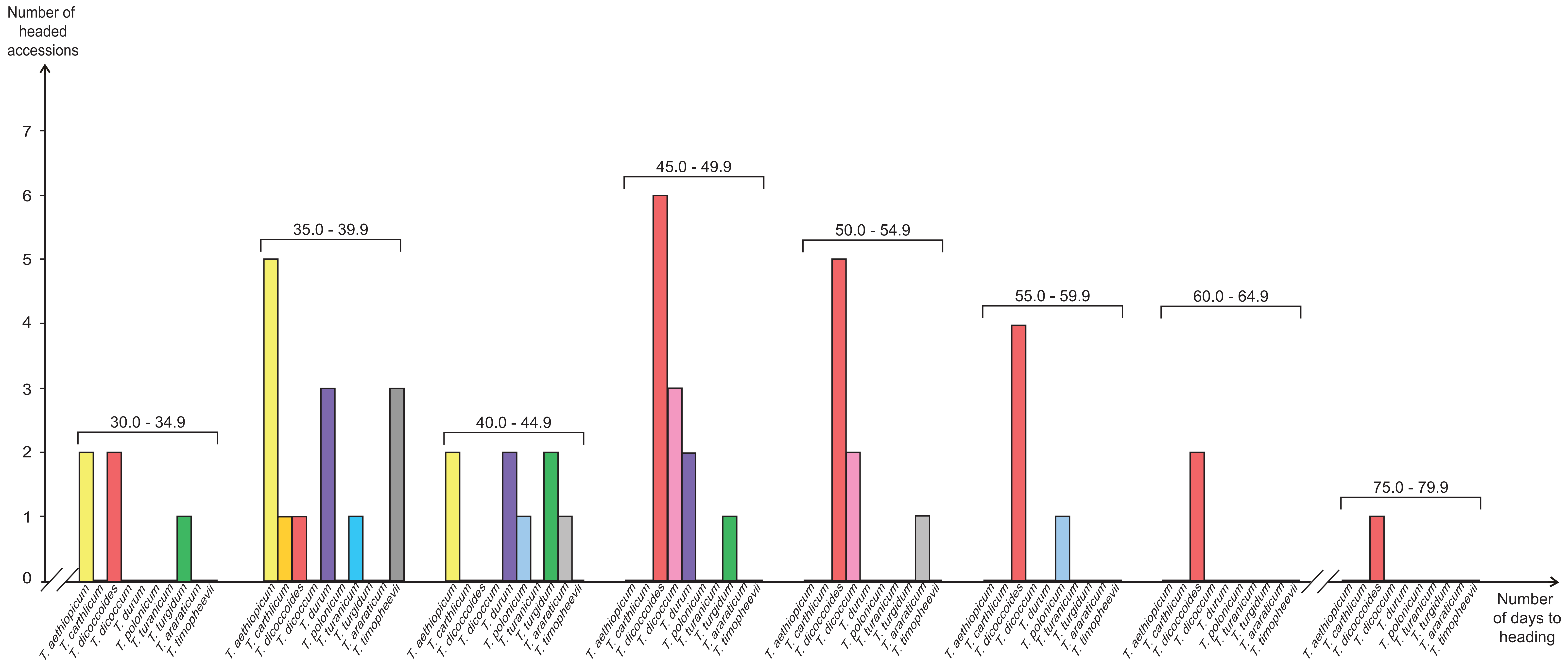


**Figure S3**. Distribution of heading time among studied accessions of ten tetraploid wheat species.
Different species are marked by different colors.

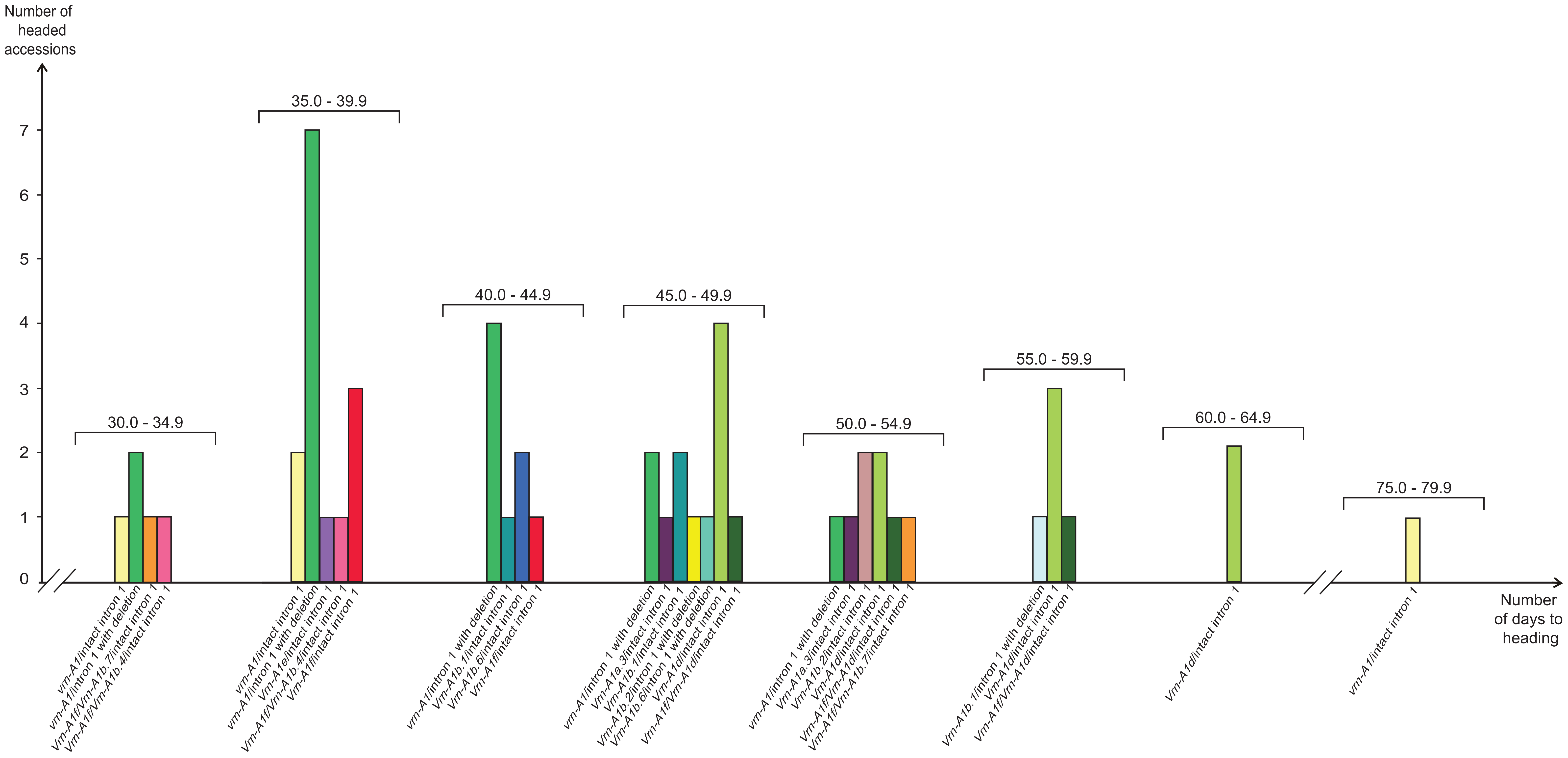


**Figure S4**. Distribution of heading time among *VRN-A1* alleles identified in studied accessions of ten tetraploid wheat species. Different allelic variants are marked by different colors.
